# Repression of *miR-29* via MYC leads to increased CD40 signaling in transformed follicular lymphoma

**DOI:** 10.1038/s41375-026-02868-8

**Published:** 2026-02-19

**Authors:** Daniel Filip, Katerina Litzmanova, Androniki Michaelou, Filip Kledus, Jan Devan, Miroslav Boudny, Eva Hoferkova, Sonali Sharma, Vaclav Seda, Pedro Faria Zeni, Marek Borsky, Kvetoslava Matulova, Leos Kren, Jan Oppelt, Nicolas Blavet, Vaclav Hejret, Milan Urik, Andrea Mareckova, Lisa M. Rimsza, Katerina Kamaradova, David Belada, Alice Sykorova, Heidi Mocikova, Marek Trneny, Zuzana Prouzova, Andrew G. Evans, Alexey Danilov, Heike Horn, German Ott, Philipp Staber, Jiri Mayer, Jonathan W. Friedberg, Andrea Janikova, Marek Mraz

**Affiliations:** 1 Molecular Medicine, Central European Institute of Technology, Masaryk University, Brno, Czech Republic; 2 Dept of Internal Medicine, Hematology and Oncology, University Hospital Brno and Faculty of Medicine, Masaryk University, Brno, Czech Republic; 3 Faculty of Science, Masaryk University, Brno, Czech Republic; 4 Dept of Pathology, University Hospital Brno and Faculty of Medicine, Masaryk University, Brno, Czech Republic; 5 Bioinformatics Core Facility, Central European Institute of Technology, Masaryk University, Brno, Czech Republic; 6 Dept of Pediatric Otorhinolaryngology, University Hospital Brno and Faculty of Medicine, Masaryk University, Brno, Czech Republic; 7 Dept of Laboratory Medicine and Pathology, Mayo Clinic, Scottsdale, AZ USA; 8 The Fingerland Department of Pathology, Charles University Faculty of Medicine and University Hospital Hradec Kralove, Kralove, Czech Republic; 9 Dept of Internal Medicine and Hematology, University Hospital Kralovske Vinohrady and Third Faculty of Medicine, Charles University, Prague, Czech Republic; 10 1st Department of Medicine - Department of Hematology, General University Hospital in Prague and First Faculty of Medicine, Charles University, Prague, Czech Republic; 11 Dept of Pathology, University, Hospital Kralovske Vinohrady and Third Faculty of Medicine, Charles University, Prague, Czech Republic; 12 Wilmot Cancer Institute, University of Rochester, Rochester, NY USA; 13 Department of Hematology and Hematopoietic Cell Transplantation, City of Hope, Duarte, CA USA; 14 Dr. Margarete Fischer-Bosch-Institute of Clinical Pharmacology, Stuttgart, Germany; 15 Dept of Clinical Pathology, Robert-Bosch-Krankenhaus, Stuttgart, Germany; 16 Dept of Medicine I, Medical University of Vienna, Vienna, Austria; 17 Dept of Internal Medicine 1, Saarland University Medical School, Homburg/Saar, Germany; 18 Present Address: University of Arizona in Tucson, Tucson, AZ USA

**Keywords:** B-cell lymphoma, Cancer microenvironment

## Abstract

Follicular lymphoma (FL) patients are at risk of disease transformation to aggressive high-grade lymphoma (tFL). While several genetic alterations have been implicated in tFL, the role of microenvironmental interactions and post-transcriptional regulation by non-coding RNAs remains poorly understood. We performed the first matched profiling of mRNAs and short non-coding RNAs (miRNAs) in paired FL and tFL samples (*n* = 11 pairs). This revealed differential expression of 1,075 mRNAs and 19 miRNAs, including repression of *miR-29* family in tFL (*miR-29a/b/c*). Further analysis uncovered that MYC directly transcriptionally represses *miR-29* in tFL, resulting in the upregulation of its target TRAF4. TRAF4 upregulation contributes to CD40 signaling being strongly activated in tFL and supports malignant B-cell proliferation. Notably, this increased CD40 pathway activity in 90% of tFL and contrasted with the reduced T-cell numbers in tFL niches. Thus, the MYC-*miR-29*-TRAF4 axis and increased CD40 signaling propensity may serve as tFL cells’ adaptation to reduced numbers of CD40L+ T-cells. Moreover, lower levels of all *miR-29s(a/b/c)* were associated with shorter overall survival (OS) and progression-free survival in FL (*n* = 185), including in a multivariate analysis. Low *miR-29c* was also associated with shorter OS in a validation cohort (*n* = 92) from the first-line R-CHOP therapy clinical trial (SWOG S0016, NCT00006721).

## Introduction

Follicular lymphoma (FL) is the most common indolent lymphoma, although ~20% of cases are characterized by early disease progression and an unfavorable prognosis [1]. Moreover, FL patients are at continuous risk of 1–3% per year of histological transformation (tFL), typically to diffuse large B-cell lymphoma (DLBCL) [2]. Several genetic alterations, including MYC gain and CDKN2A/B or TP53 activity loss as well as aberrant somatic hypermutations, were shown to be involved in tFL [3–5]. Arguably, the gain in MYC activity is regularly observed in tFL (>70% of cases), however, MYC genetic aberrations are present in only a proportion of cases [3–9], suggesting MYC activation via signaling. Importantly, FL transformation is also accompanied by co-evolution of the tumor microenvironment [10, 11]. Analyzing tFL has shown that tFL cells tend to escape recognition by cytotoxic T-cells while other B-T-cell interactions remain potentially active [12, 13]. T-cells represent the most common non-malignant cell type in FL/tFL, and it has been shown that T follicular helper (Tfh) cells support FL cell proliferation and survival by activating CD40 signaling and MYC expression. Indeed, mice engineered to have constitutively high CD40L on mature T-cells develop GC lymphomas [14]. However, it remains unclear if B-T-cell interactions are important in transformed FL.

Several studies have performed gene expression profiling on FL-tFL samples [6, 8, 15–18], however, the results are partially discordant. Moreover, these studies have not included the matched analysis of post-transcriptional regulation by short non-coding RNAs, microRNAs. MicroRNAs (miRNAs) can post-transcriptionally regulate mRNA expression by binding to their 3’UTR and thus tune essential cell functions such as B-cell development and survival, B-cell receptor signaling, and lymphomagenesis in mouse models [19]. We and others have shown that *miR-150* and its target FOXP1 are associated with tFL development, and FL aggressiveness and BCR signaling activity [9, 11, 19–23]. However, it remains unclear if miRNAs can contribute to FL transformation by regulating microenvironmental interactions.

Here we performed the first matched profiling of miRNAs and mRNAs in paired FL and tFL samples (*n* = 11 pairs). This identified differential expression of 1075 mRNAs and 19 miRNAs in tFL, including *miR-29a/b/c* repression. Further analysis revealed that in tFL, MYC transcriptionally represses *miR-29*, resulting in upregulation of its target Tumor Necrosis Factor Receptor-Associated Factor 4 (TRAF4), leading to enhanced CD40 signaling propensity during B-T cell interactions and malignant B-cell proliferation. These findings reveal for the first time the increase in CD40 signaling in the great majority of tFL (~90%) and the role of *miR-29* in this process. Notably, T-cell content is generally reduced in tFL, suggesting that this pathway represents a tFL cell adaptation to relatively lower access to CD40L+ T-cells. Moreover, we have shown that *miR-29s* can be used as biomarkers of unfavorable FL prognosis.

## Methods

### Patient samples and cell lines

Eleven pairs of clonally related FL-tFL samples (Formalin-fixed paraffin-embedded [FFPE]) were analyzed (*n* = 22) by mRNA-seq and 10 FL-tFL pairs were analyzed by miRNA-seq (9 pairs overlapping with mRNA-seq) (Supplementary Table 1). All the analyzed tFLs were clonally related and histologically verified transformations to DLBCL. For testing of miRNAs as biomarkers in FL we utilized a discovery cohort (*n* = 185, FFPE samples), validation cohort from the R-CHOP arm of the S0016 trial (NCT00006721 [24, 25]; *n* = 92 available FFPE samples), and DLBCL FFPE samples (*n* = 174); cohorts characteristics in Supplementary Tables 2–4. Additional FL-tFL pairs (*n* = 10 pairs), tFL (*n* = 15), FL (*n* = 94) and DLBCL (*n* = 30) samples were used in additional analysis. SU-DHL4, KARPAS422, DOHH2, WSU-NHL, HEK293-FT, and HS5 cell lines were obtained from DSMZ or ATCC and cultured (5% CO_2_, 37 °C) in recommended media with 10% FBS (Biosera) and 100 U·ml^−1^/100 μg·ml^−1^ penicillin/streptomycin (Sigma Aldrich). For functional studies and experimental procedures, see supplemental Methods.

### miRNA and mRNA profiling

Twenty FFPE samples (10 FL-tFL pairs; Supplementary Table 1) were used for library preparation with NEBNext Multiplex Small RNA Library Prep Set (NEB) for Illumina, according to the manufacturer’s protocol. Briefly, 800 ng of RNA were used as input, and the libraries sequenced using NextSeq (Illumina). For mRNA analysis, 22 FFPE samples (11 FL-tFL pairs, overlapping with miRNA-seq; Supplementary Table 1) were used for library preparation (QuantSeq 3’ mRNA-seq Library Prep Kit FWD, Lexogen). For details on library preparation, data analysis and experimental procedures, see supplemental Methods. The data have been deposited to EGA (EGAD50000001384 for mRNA-seq and EGAD50000001385 for miRNA-seq).

### Public data acquisition

scRNA-seq data (10× Genomics) and cell annotation from Roider et al. [26] (10.11588/data/VRJUNV) were processed by CellRanger (3 reactive lymph nodes, 4 FL, 3 DLBCL, 2 tFLs). RNA-seq data from Parsa et al. [18]. (GEO:GSE142334) generated from 6 paired FL-tFL frozen lymph node biopsies were analyzed using limma (v3.54.2) by performing batch correction for pairs and differential expression analysis. Expression data from 149 FL patients from Huet et al. [27] (GEO:GSE93261) from were analyzed using limma (v3.54.2).

### Statistical analysis

Apart from NGS data analysis (see supplemental Methods), statistical analyses were performed with GraphPad Prism v5.0 and Statistica 13.2 (TIBCO Software Inc.). *P*-values < 0.05 were considered significant.

## Results

### *miR-29* family is downregulated upon FL transformation

**Fig. 1:**
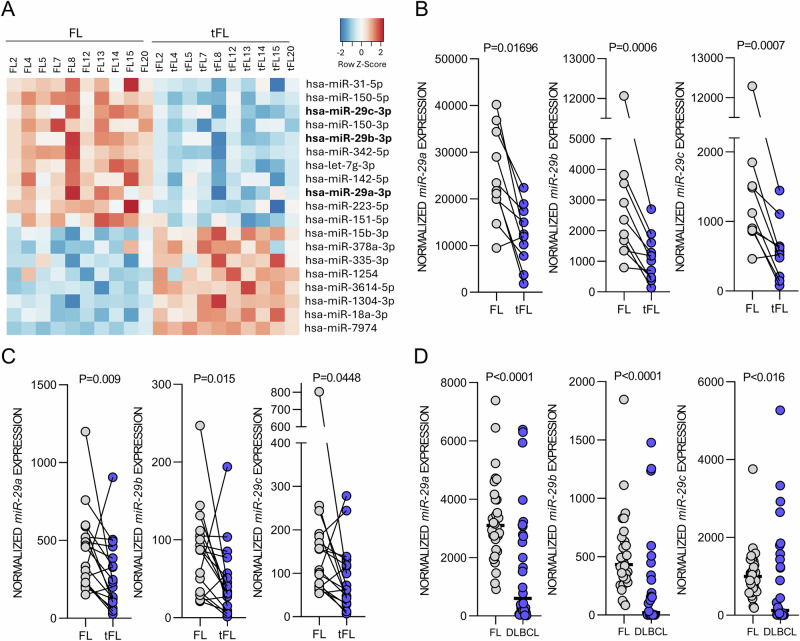
*miR-29* family is downregulated in tFL. **A** Heatmap of differentially expressed miRNAs (*P*adj<0.05, fold-change >1.5) in paired FL-tFL samples (*n* = 10 pairs). Row z-score from normalized counts for each miRNA was plotted. Lower expression is indicated in blue, and higher expression in red. All tFLs were histologically verified as DLBCL. All RNA samples in the analysis were isolated from FFPE tissue. **B** Normalized expression of *miR-29(a/b/c)* from miRNA-seq of paired FL-tFL samples (*n* = 10 pairs). **C** Validation of *miR-29a/b/c* levels in paired FL-tFL (*n* = 17 pairs). The same 10 pairs from panel A and additional 7 pairs (14 samples) were analyzed by qRT-PCR. Statistical differences were compared by Wilcoxon matched paired test. **D** *miR-29a/b/c* expression assessed by qRT-PCR in FL (*n* = 30) and de novo DLBCL (*n* = 30) samples. Statistical differences were compared by Mann–Whitney test.

In our previous study, we utilized qRT-PCR technology to analyze 377 selected miRNAs in paired FL and tFL samples, identifying 5 differentially expressed miRNAs [9]. Here we performed the first profiling of all human miRNAs in 10 FL-tFL pairs (*n* = 20), revealing 19 miRNAs with significantly changed expression in tFL (*P*adj <0.05, fold-change >1.5; Fig. 1A). This included miRNAs identified in the previous qRT-PCR screen [9] (*miR-150-5p*, *miR-31*-*5p*) and notably downmodulation of all three members of the *miR-29* family (*miR-29a/b/c)* in tFL (*miR-29a* fold-change = 2.0, *miR-29b* fold-change = 2.36, *miR-29c* fold-change = 2.51; Fig. 1B). We and others have previously shown that *miR-29s* regulate the aggressiveness of CLL and other B-cell malignancies [28–30], and we decided to further focus on this miRNA family. Validation (qRT-PCR) in a larger cohort of 17 FL-tFL pairs confirmed significant *miR-29s* down-regulation in tFL (Fig. 1C), and lower *miR-29s* expression in de novo DLBCL samples (*n* = 30) than FL (*n* = 30) (Fig. 1D). Altogether, *miR-29* family is downregulated in tFL, which might have important consequences as *miR-29s* potentially regulate a number of processes in B-cells.

### tFL cells have increased CD40 signaling

**Fig. 2:**
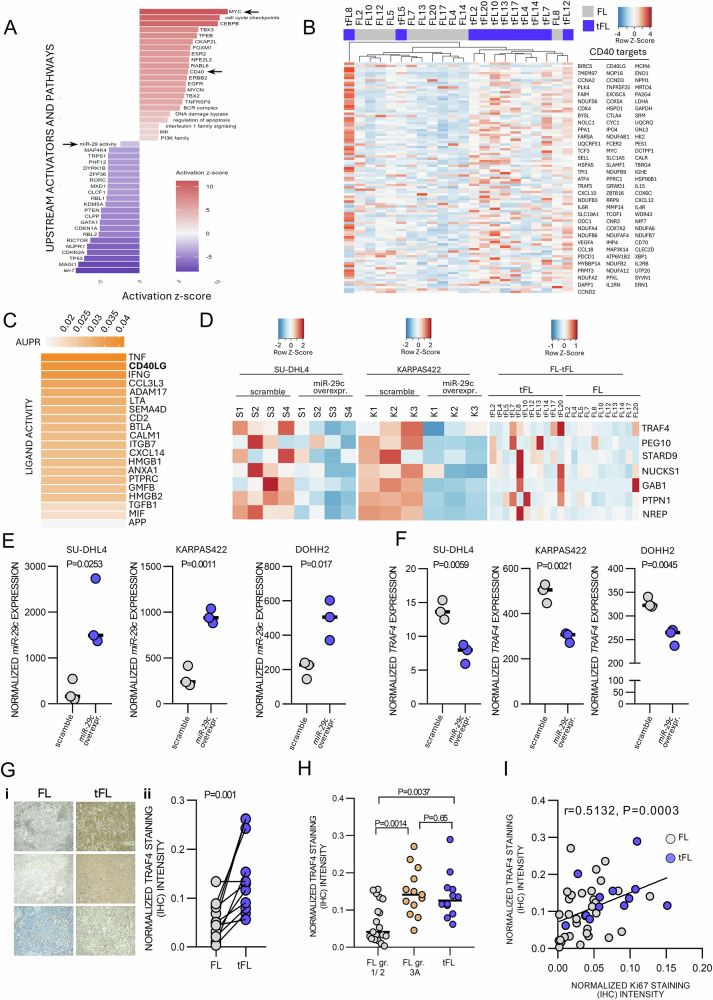
CD40 signaling is increased in tFL and *miR-29* regulates *TRAF4* levels. **A** Analysis of upstream regulators and pathways (IPA) from mRNA-seq of FL-tFL pairs (*n* = 11). Red color represents increased activity of upstream regulator/pathway, blue color represents decreased activity. **B** Unsupervised clustering of FL and tFL samples (*n* = 11 pairs) using expression of mRNAs annotated as CD40 pathway targets (*n* = 98; mRNAs identified by IPA). Row z-score from normalized counts for each mRNA was plotted. Lower expression indicated in blue, and higher expression in red. Blue color in the legend represents tFL samples while gray color represents FL samples. **C** NicheNet analysis of microenvironment ligands responsible for gene expression differences between FL and tFL samples from Roider et al. [26] scRNA-seq data. B-cells were used as receiver cells and T helper, Tfh, T regulatory, and T cytotoxic cells were used as sender cells. Ligands are ranked based on the area under the precision-recall curve (AUPR) between a ligand’s target predictions and the observed transcriptional response. The stronger the orange color, the stronger the effect of ligand on gene expression differences between FL and tFL cells. **D** Heatmap of seven anti-correlated *miR-29* targets significantly downregulated (*P*adj < 0.05) in both SU-DHL4 and KARPAS422 cell lines over-expressing *miR-29c* (left and middle section). Expression of these seven *miR-29* targets was also visualized in 11 FL-tFL pairs (mRNA-seq, right section). Row z-score from normalized counts for each mRNA was plotted. Lower expression indicated in blue, and higher expression in red. *miR-29c* expression in control (scramble) and *miR-29c* overexpressing SU-DHL4, KARPAS422 and DOHH2 (qRT-PCR, *n* = 3). Statistical differences were compared by *t* test. **E** *miR-29c* expression in control (scramble) and *miR-29c* overexpressing SU-DHL4, KARPAS422 and DOHH2 (qRT-PCR, *n* = 3). Statistical differences were compared by *t* test. **F** *TRAF4* mRNA expression in control (scramble) and *miR-29c* overexpressing SU-DHL4, KARPAS422 and DOHH2 cell lines (qRT-PCR, *n* = 3). Statistical differences were compared by *t* test. **G** Representative TRAF4 staining (IHC) in 11 FL-tFL pairs (i) and staining quantification performed by ImageJ FIJI (ii). Statistical differences were compared by Wilcoxon matched paired test (**H**) Quantification of TRAF4 staining (IHC) performed by ImageJ FIJI in FL grade 1 and 2 (*n* = 19), FL grade 3 (*n* = 13), and tFL (*n* = 12) samples. Statistical differences were compared between each group by Mann–Whitney test. **I** Correlation of TRAF4 and Ki67 staining quantification (IHC) performed by ImageJ FIJI in FL/tFL samples (*n* = 46; Spearman correlation).

Each individual miRNA can bind and regulate a number of possible mRNAs based on their complementarity and specific cellular context. The *miR-29* family has 1265 predicted evolutionarily conserved targets (TargetScan tool) and multiple mRNAs have been identified as being regulated by *miR-29s* in various cell types including B-cells [29–31]. However, the genes targeted by a certain miRNA can vary depending upon the cellular context including in each B-cell maturation stage [32]. Because a miRNA can influence the relative stability of its target mRNA [23, 33], evaluating for differences in gene expression between FL and tFL cells could identify mRNA(s) that are anti-correlated to *miR-29s* levels and might be potentially regulated by this miRNA. As such, and to better understand transcriptional changes during FL transformation, we performed RNA profiling of 11 paired FL-tFL FFPE samples (*n* = 22; 9 pairs overlapping with miRNA-seq above). This identified 1,075 differentially expressed mRNAs (*P*adj < 0.05, fold-change >1.5; Supplementary Fig. S1A; PCA in Supplementary Fig. S1B), and to our knowledge, this is the first time both mRNA and miRNA transcriptomes have been analyzed in FL-tFL pairs. Subsequent GSEA analysis revealed changes in multiple signaling pathways in tFL, including gain of MYC and E2F activity, induction of Oxidative phosphorylation and mTORC1 activity (Supplementary Fig. S1C–E). We also utilized IPA tool to identify upstream regulators and pathways changed in tFL, and in line with reduced *miR-29s* levels, the tFL cells had significantly higher levels of mRNAs putatively repressed by *miR-29s* (Fig. 2A). Additionally, RNA-seq in FL-tFL pairs revealed changes in multiple molecular pathways potentially controlled by *miR-29s*, including CD40 signaling being strongly activated in tFL (Fig. 2A), which was also reflected in the activation of downstream NF-kB pathway (Supplementary Fig. S2A, B). This caught our attention since CD40 is a prominent driver of B-cell proliferation. Unsupervised clustering of FL and tFL samples revealed that a majority of tFL samples (10 out of 11) have an increased CD40 signaling signature (Fig. 2B). The CD40 pathway activation was also confirmed in public transcriptomes from FL-tFL pairs [18] (*n* = 6; Supplementary Figs. S3, S4). Single-cell RNA-seq data [26] revealed that CD40L is mostly expressed by Tfh cells (Supplementary Figs. S5, S6), but could be potentially also provided by macrophages with low-level CD40L expression [34] that would be difficult to detect by scRNAseq. Notably, CD40L was amongst the top 10 most active ligands in tFL in NicheNet analysis (Fig. 2C), with almost all CD40L transcriptional targets being expressed more in tFL than FL (Supplementary Fig. S7). This observation was unexpected given the typically reduced T-cell numbers in tFL (ref. [12], and see below). Altogether, this data shows for the first time that CD40 signaling is very frequently enhanced in tFL, and we hypothesized that *miR-29s* play a role in this process.

### *miR-29* represses TRAF4 levels and CD40 signaling

**Fig. 3:**
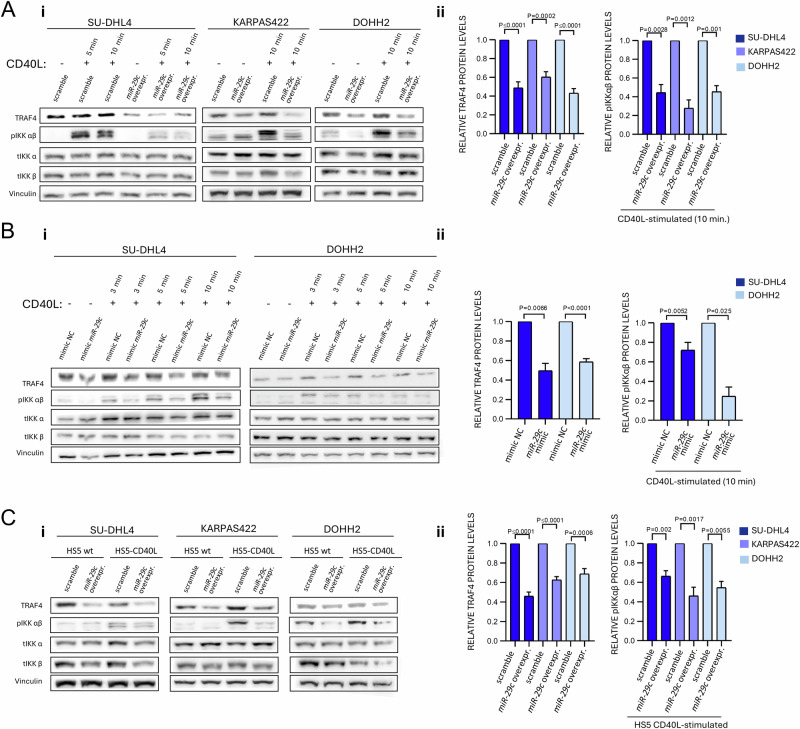
*miR-29* and *TRAF4* levels determine CD40 signaling propensity. **A** (i) Representative immunoblot analysis of SU-DHL4, KARPAS422, and DOHH2 with constitutive expression of control (scramble) or *miR-29c*, and treated with CD40L (1 ug/ml, 5 or 10 min.). Vinculin was used as a loading control. (ii) Densitometric quantification of TRAF4 levels (SU-DHL4 *n* = 9, KARPAS 422 *n* = 8, DOHH2 *n* = 7) and pIKK levels in CD40L-stimulated samples from [A] (SU-DHL4 *n* = 5, KARPAS 422 *n* = 5, DOHH2 *n* = 5). **B** (i) Representative immunoblot analysis of SU-DHL4 and DOHH2 cells transfected with miRNA mimic negative control (mimic NC) or *miR-29c* mimic. Transfected cells were stimulated with CD40L (1 μg/ml, 3–10 min.). Vinculin was used as a loading control. (ii) Densitometric quantification of TRAF4 levels (SU-DHL4 *miR-29c* mimic *n* = 4, DOHH2 mimic *n* = 5) and pIKK levels in CD40L-stimulated samples (SU-DHL4 *miR-29c* mimic *n* = 3, DOHH2 mimic *n* = 3). **C** (i) Representative immunoblot of SU-DHL4, KARPAS422, and DOHH2 cells with constitutive expression of control (scramble) or *miR-29c* after co-culture with HS5 wt or HS5-CD40L expressing cells (6 h). Vinculin was used as a loading control. (ii) Densitometric quantification of TRAF4 levels (SU-DHL4 *n* = 10, KARPAS422 *n* = 8, DOHH2 *n* = 8) and pIKK levels in HS5-CD40L-cocultured samples (SU-DHL4 *n* = 6, KARPAS422 *n* = 6, DOHH2 *n* = 4). The statistical differences in all quantification figures were tested by paired *t* test, and the error bars indicate the standard error of the mean (SEM).

To directly describe the effects of *miR-29* on B-cell transcriptome, we performed RNA profiling in 2 cell lines engineered for *miR-29c* overexpression (SU-DHL4 and KARPAS422). We selected *miR-29c* for these experiments since all the *miR-29* family members have an identical seed sequence, and *miR-29c* had the strongest downregulation of the whole *miR-29* family in tFL (fold change=2,51; Fig. 1B). This revealed 861 and 2541 differentially expressed mRNAs (*P*adj < 0.05) in SU-DHL4 and KARPAS422 cells with ~6.9- and ~3.3-fold *miR-29c* overexpression, respectively. This included 57 and 137 downregulated mRNAs in SU-DHL4 and KARPAS422 with evolutionary conserved *miR-29* binding sites, respectively, and 20 such mRNAs overlapped in both cell lines. Notably these twenty *miR-29* targets were able to significantly segregate FL patients (*n* = 149) [27] into groups with relatively good vs. worse prognosis (*P* = 1.7^-08^; Supplementary Fig. S8). To identify the *miR-29* targets most relevant for tFL, we plotted the expression of these 20 mRNAs also in paired FL-tFL samples and noted a trend for anti-correlation (higher mRNA levels) of 7 mRNAs in tFL (Fig. 2D). Considering the increased CD40 signaling activity in tFL (see above), we selected TRAF4 from these analysis for further studies. TRAF4 is the only target from the 20 mRNAs linked to CD40 signaling [29], and *miR-29c* represses *TRAF4* via binding to its 3’UTR (Supplementary Fig. S9A). However, the binding and regulation of a given mRNA by specific miRNA depends on their stochiometric ratio, presence of other targets of the same miRNA that can sponge it, and contributions of other regulators of target mRNA translation/stability (including other miRNAs) [19, 35, 36]. To validate that *miR-29* affects TRAF4 levels, we analyzed 3 lymphoma cell lines with *miR-29c* overexpression (SU-DHL4 and KARPAS422 used for mRNA-seq, and DOHH2). These DLBCL cell lines were selected for harbouring BCL2 translocation (14;18), a hallmark of FL and clonally related tFL. This revealed that moderate *miR-29c* overexpression leads to statistically significant ~50% down-modulation of *TRAF4* mRNA (Fig. 2E, F) and protein levels (see below Fig. 3A). Next, we tested if TRAF4 levels are higher in tFL in vivo. Indeed, TRAF4 levels (IHC) were increased in tFL compared to paired FL (*n* = 11 pairs; Fig. 2G) and in de novo DLBCL compared to FL (*n* = 30 each, Supplementary Fig. S9B). Similarly, scRNA-seq data [26] indicate that *TRAF4* expression is stronger in tFL compared to FL (Supplementary Fig. S10). Moreover, TRAF4 protein levels were higher in tFL samples (*n* = 23) and grade 3A FL (*n* = 17) than grade 1-2 FL (*n* = 30), underscoring the possible relevance for FL cell aggressiveness (Fig. 2H). Indeed, TRAF4 levels (IHC) positively correlate with Ki67 (cell proliferation marker) and Ki67 negatively correlated with *miR-29s* levels (Fig. 2I, Supplementary Fig. S11).

Next, we tested the effect of *miR-29* on CD40 signaling and observed that the 3 cell lines engineered for constitutively overexpressing *miR-29c* (SU-DHL4, KARPAS422, DOHH2) were less responsive to recombinant CD40L as evidenced by lower IKKa/b phosphorylation (Fig. 3A). The phenotype resembled decreased CD40 responsiveness in cells transfected with siRNA against *TRAF4* or synthetic *miR-29c* mimic (Fig. 3B, Supplementary Fig. S12A). On the other hand, overexpression of TRAF4 (from a construct not containing its 3’UTR with miRNA binding site) led to an increased IKKa/b phosphorylation after CD40L and rescued the negative effect of *miR-29* on CD40 signaling propensity (Supplementary Fig. S12B). The *miR-29c* overexpressing cell lines were also less responsive to a cell-membrane CD40L presented by HS5 cells, which better resembles the in vivo interaction with T-cells [37, 38] (Fig. 3C). Additionally, the transfection of primary FL cells with synthetic *miR-29* also reduced their proliferation rate in response to co-culture with HS5-CD40L-IL4-IL21 cells (Supplementary Fig. S13), a model that robustly induces CD40L-triggered proliferation of malignant B-cells [38]. Altogether, this demonstrates that *miR-29* downmodulation in tFL results in higher TRAF4 levels and increased CD40L signaling propensity.

### T-cell populations are reduced in tFL

**Fig. 4:**
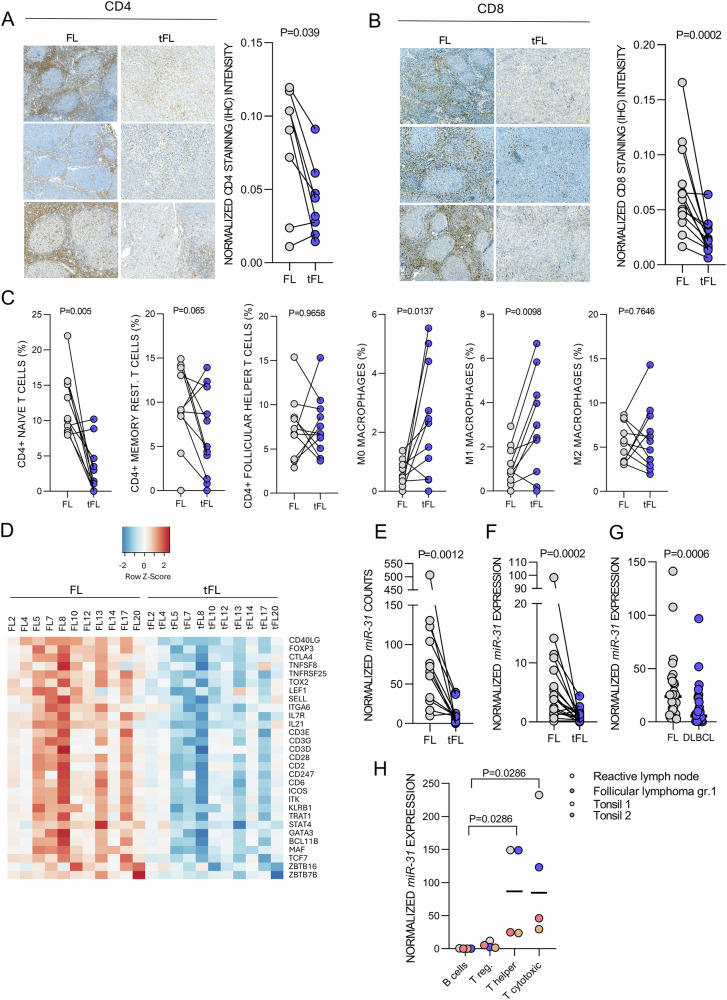
T-cell content is reduced in tFL. Representative staining of CD4 + T cells (**A**) and CD8 + T-cells (**B**) in FL-tFL pairs (CD4: *n* = 6 pairs; CD8: *n* = 10 pairs) and FL/tFL composite samples (CD4: *n* = 2; CD8: *n* = 3; FL vs. tFL component analyzed separately). Staining quantification performed by ImageJ FIJI on the right. Statistical differences were compared by Wilcoxon matched paired test. **C** The CIBERSORTx analysis of the abundance of selected T cell and macrophage populations from FL-tFL pairs (bulk mRNA-seq, *n* = 11). Statistical differences were compared by Wilcoxon matched paired test. Remaining cell populations are depicted in Supplementary Figs. S14, S15. **D** Expression of T-cell specific gene markers (signature of *n* = 29 mRNAs) in 11 FL-tFL pairs (mRNA-seq). Row z-score from normalized counts for each mRNA was plotted. Lower expression indicated in blue, and higher expression in red. **E** *miR-31* normalized expression in 10 FL-tFL pairs (miRNA-seq counts). **F** *miR-31* expression in FL-tFL pairs (*n* = 19 pairs). The same 10 pairs from Fig. 1A and additional 9 pairs (18 samples) were analyzed by qRT-PCR. Statistical differences were compared by Wilcoxon matched paired test. **G** *miR-31* expression (qRT-PCR) in an independent cohort of FL (*n* = 30) and de novo DLBCL (*n* = 30) samples. Statistical differences were compared by the Mann–Whitney test. **H** *miR-31* expression (qRT-PCR) in different T-cell populations sorted from samples of a fresh healthy lymph node (*n* = 1), two control tonsils (*n* = 2), and FL lymph node (*n* = 1, FL grade 1). Cells were sorted according to the following markers: B-cells (CD19+), T regulatory cells (CD4+CD25+), T helper cells (CD4+CD25−), and T cytotoxic cells (CD8+). Statistical differences were compared by Mann–Whitney test.

The CD40 signaling upregulation upon FL transformation led us to analyze T-cell content in FL/tFL. The IHC staining showed that the amount of both CD4+ and CD8 + T-cells decreased in tFL (Fig. 4A, B). The in silico cell population analysis (CIBERSORTx) from our bulk RNA-seq identified complex changes in cell populations in tFL (Supplementary Figs. S14, S15) including reduced memory naïve and resting CD4+ T-cells, while macrophage content (M0/1) increased in tFL (Fig. 4C). T-cell signature genes and processes such as TCR signaling were reduced in tFL (Fig. 4D, Supplementary Figs. S16, S17). Notably, the content of Tfh cells (a dominant CD40L source) seemed unchanged in tFL based on CIBERSORTx (Fig. 4C). Generally decreased T-cell content is concordant with DLBCL transformation leading to disrupted GC architecture with a higher malignant B-cell percentage and engagement of T cell evasion mechanism [8, 12, 39–43]. In line with reduced T-cell numbers, we noted downmodulation of a T-cell specific *miR-31* [44, 45] in tFL (Figs. 1A and 4E–G). Indeed, sorting of B/T-cell populations from reactive tonsils, lymph nodes, and FL lymph node, showed that *miR-31* is not detectable in B-cells, but is expressed primarily in CD4 + T helper and most strongly in CD8+ T-cells (Fig. 4H), that are consistently depleted in tFL niches (Fig. 4B). Moreover, T-cell content and *miR-31* expression were strongly positively correlated in FL/tFL (Supplementary Fig. S18) suggesting that lower *miR-31* expression in tFL is a surrogate marker for reduced T-cell numbers and/or activity. Altogether, these data suggest that the increase in CD40 signaling in tFL cells is more likely a cell-intrinsic event and is not due to an increase in CD40L availability via increased T-cell quantities.

### Increased MYC activity represses *miR-29* during FL transformation

**Fig. 5:**
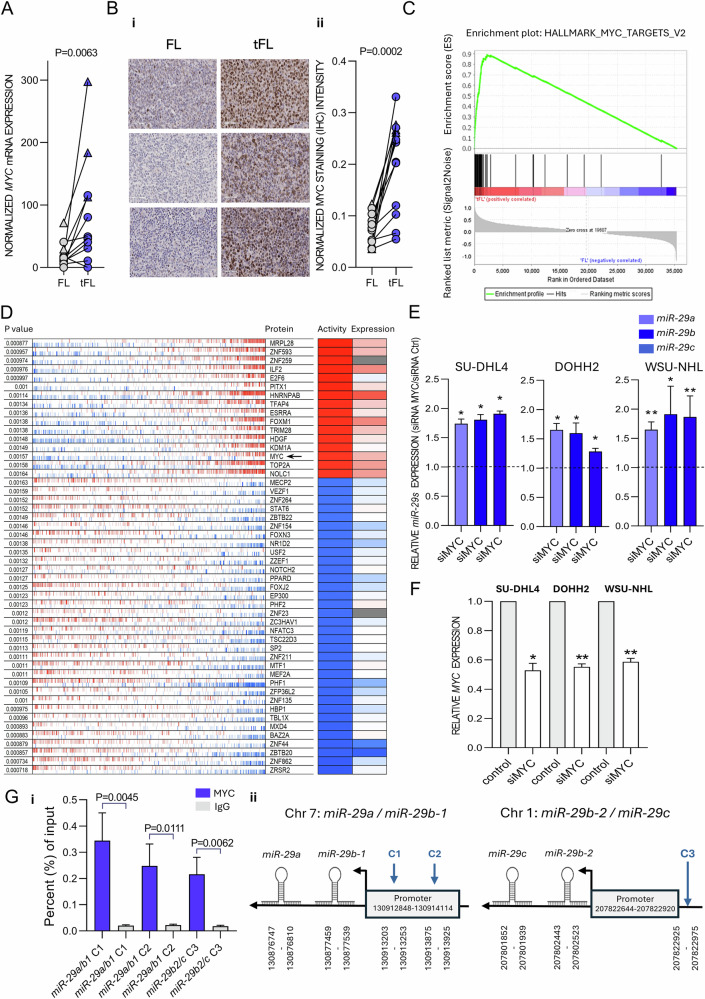
MYC acts as a repressor of *miR-29* family in tFL. **A** Normalized *MYC* mRNA levels in FL-tFL pairs (*n* = 11 pairs). Samples with MYC translocation are visualized as triangles. **B** Representative IHC staining of MYC in 13 FL-tFL pairs (i) and staining quantification performed by ImageJ FIJI (ii). Original magnification ×400. Statistical differences were compared by Wilcoxon matched paired test. Samples with MYC translocation are visualized as triangles. **C** GSEA enrichment analysis from mRNA-seq of FL-tFL pairs (*n* = 11) indicating strong enrichment of MYC targets (HALLMARK_MYC_TARGETS_V2 gene set; NES = 1.93, FDR = 0.016). **D** VIPER analysis of mRNA-seq of FL-tFL pairs (*n* = 11) depicting top 50 proteins with change in activity in tFL. Red color represents increased activity, blue color represents decreased activity. Relative expression of *miR-29a/b/c* (qRT-PCR, **E**) or *MYC* (qRT-PCR, **F**) in SU-DHL4, DOHH2 and WSU-NHL cells transfected with siRNA against *MYC* (SU-DHL4 and DOHH2: *n* = 3, WSU-NHL: *n* = 4). Statistical differences were compared by paired *t* test. **G** (i) Chromatin immunoprecipitation analysis of 3 putative evolutionary conserved MYC binding sites in *miR-29s* promoter. Two regions for MYC binding in the promoter of *miR-29a/miR-29b-1* were tested (C1: chr 7: position 130913203-130913253; C2: chr 7: position 130913875-130913925; GRCh38/hg38), and one site near *miR-29b-2/miR-29c* promotor was tested (C3: chr 1: position 207822925-207822975, GRCh38/hg38). The qRT-PCR results are represented as the DNA input percentage, and error bars indicate the standard error of the mean. Rabbit IgG antibody was used as control. (ii) Graphical depiction of MYC binding sites in promoter regions of the *miR-29a/miR-29b-1* on chromosome 7 and *miR-29b-2/miR-29c* on chromosome 1 (genomic positions indicated, human genome assembly version GRCh38/hg38).

We next aimed to identify the mechanism for *miR-29s* downmodulation in tFL since *miR-29s* can be regulated by a number of transcription factors [29, 46, 47]. The analysis of the database of the known *miR-29*s transcriptional regulators (TransmiR v3.0 tool) revealed that in FL-tFL RNA-seq data, *MYC* is the most differentially expressed repressor of *miR-29s* in tFL (Fig. 5A, Supplementary Fig. S19). Indeed, MYC protein levels were induced in all analyzed tFL cases (IHC staining of 13 FL-tFL pairs; 8 available FL-tFL pairs used for RNA-seq and 5 additional pairs; Fig. 5B). In only 3 of these 13 tFL samples, we detected MYC translocation, hinting to signaling events inducing MYC activity. The transcriptomic data confirmed strong MYC activity (GSEA) enrichment in tFL (Fig. 5C, Supplementary Figs. S1C, S20). Transcription factor (TF) activity analysis using 3 different prediction tools validated this, identifying MYC as one of the most active TFs upon FL transformation (Figs. 2A, 5D, Supplementary Fig. S21). Gaining MYC activity in tFL was also observed in our analysis of a publicly available dataset of 6 paired FL-tFL (fresh-frozen) samples [18] (Supplementary Figs. S3, S22, S23) and single-cell RNA-seq data [26] (Supplementary Fig. S24). To test MYC’s direct role in *miR-29* regulation, we transfected 3 lymphoma cell lines with siRNA against *MYC*, revealing significant upregulation of all *miR-29s* (Fig. 5E, F). The co-regulation of all *miR-29* family members (encoded by two miRNA clusters) is supported by their generally highly correlated expression (Supplementary Fig. S25A–C). To test for binding of MYC to the *miR-29s* promoter, we performed ChIP-qPCR in SU-DHL4 lymphoma cell line using primers for 3 regions containing predicted evolutionary conserved MYC binding sites in *miR-29* promotors (two sites in *miR-29a/b*, one site in *miR-29b/c* promotor) [46]. This revealed MYC binding at all 3 *miR-29s* promoter regions (Fig. 5G), which supports a direct transcriptional repression of *miR-29s* by MYC. In conclusion, this underscores consistently increased MYC activity in tFL independently of MYC translocation and demonstrates that MYC represses *miR-29s*.

### Low *miR-29s* levels are associated with unfavorable FL prognosis

**Fig. 6:**
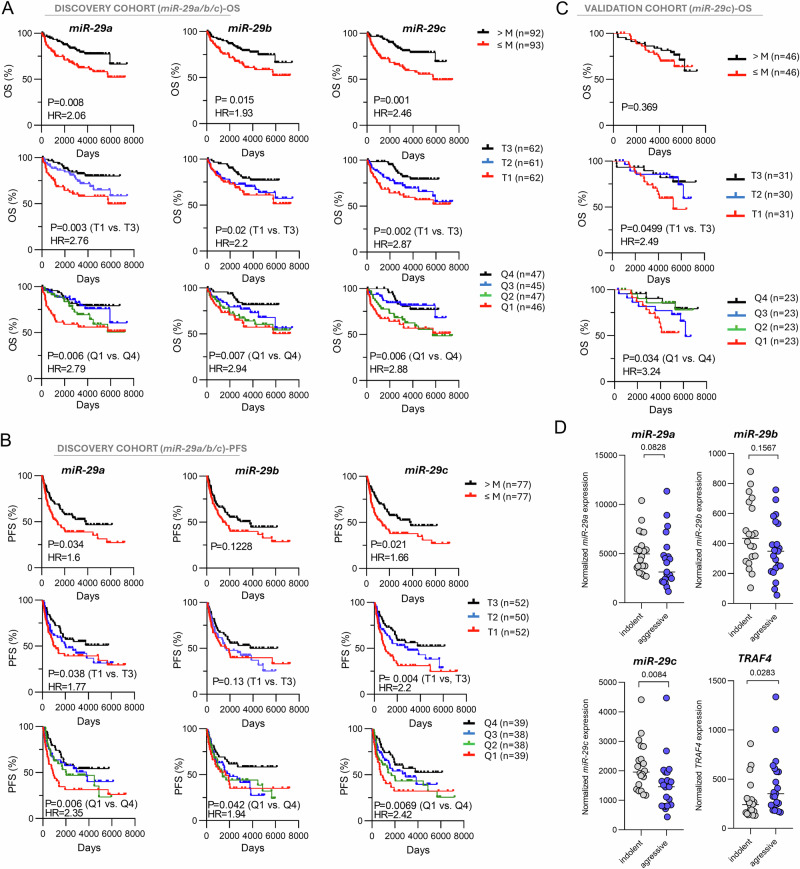
*miR-29* family is associated with prognosis and aggressivity in FL. **A** Association of *miR-29a/b/c* with OS in FL patients (*n* = 185). The cohort included FFPE samples obtained at diagnosis (*n* = 154) and at progression (*n* = 31). The *miR-29a* expression level was not available for 1 sample because of technical issues with its quantification. Patients were separated based on the median (M), tercile (T1–3), and quartile (Q1–4) of miRNA expression. For association with OS in a cohort of only FL samples obtained at diagnosis (*n* = 154 out of 185) see Supplementary Fig. S26. Expression of *miR-29a/b/c* was normalized to expression of *RNU38B*. **B** Association of *miR-29a/b/c* expression with PFS in FL patients (only samples obtained at diagnosis [*n* = 152 out of 183] were included in the analysis). All FFPE samples were obtained at diagnosis, and patients were separated based on the median (M), tercile (T1–3), and quartile (Q1–4) of miR expression. Expression level of *miR-29a* was not available for 1 sample because of technical issues with its quantification. Expression of *miR-29a/b/c* was normalized to expression of *RNU38B*. **C** Association of *miR-29c* expression with OS in a validation FL cohort (*n* = 92) uniformly treated by R-CHOP (trial SWOG S0016). Patients were separated based on the median (M), tercile (T1–3), and quartile (Q1–4) of *miR-29c* expression. For *miR-29a/b* see Supplementary Fig. S27. Expression of *miR-29c* was normalized to geometric mean of *RNU38B, RNU6B*, and *miR-16* expression (3 endogenous controls used to increase robustness). **D** Expression levels of *miR-29a, miR-29b, miR-29c*, and TRAF4 in fresh frozen samples from FL patients with aggressive (*n* = 21) or indolent (*n* = 20) course of disease. Aggressive patients were defined as patients who experienced disease progression within 3 years from the start of therapy. Indolent patients were defined as patients who did not progress for more than 8 years from the start of therapy (only 2 out of 20 patients progressed during follow-up after 8 and 13 years, respectively [median follow up: 11 years, range: 7–16 years]). Statistical differences were compared using the Mann–Whitney test. Only grade 1–3A was considered as FL in our study.

*miR-29s* affect CD40 signaling during FL transformation, but *miR-29* and its targets might also be relevant for non-transformed FL. Indeed, lower levels of all *miR-29s(a/b/c)* were associated with shorter OS (*n* = 185) and PFS in FL (Fig. 6A, B, Supplementary Fig. S26). Among *miR-29s*, the *miR-29c* was the strongest predictor of shorter OS and PFS (median OS for 1st vs. 3rd tercile: 6.6 vs. 10.4 years, *P* = 0.002; HR: 2.9 [CI: 1.5–5.6]; median PFS for 1st vs. 3rd tercile: 2.2 vs. 6.6 years, *P* = 0.004; HR: 2.2 [CI: 1.3–3.7). The association of *miR-29a/b/c* with FL prognosis was also significant in a multivariate analysis, which included several known prognostic markers (age, FLIPI, hemoglobin levels, LDH levels, Ann Arbor stage, presence of B symptoms; Supplementary Table 5). We next attempted to validate these results in an independent cohort of FFPE samples (*n* = 92) from an R-CHOP first-line therapy clinical trial (NCT00006721 [24], Supplementary Table 3). Lower *miR-29c* expression was the only *miR-29* associated with shorter OS in this validation cohort (Fig. 6C, Supplementary Fig. S27), and this was significant also in a multivariate analysis (Supplementary Table 6). Moreover, combining *miR-29c* expression with FLIPI identified patients with the shorter OS within FLIPI groups in both discovery and validation cohort (Supplementary Fig. S28). The lower *miR-29a*/*b*/*c* levels’ association with PFS was not statistically significant in the validation cohort (Supplementary Fig. S29). However, lower *miR-29c* levels and high TRAF4 levels were associated with an aggressive disease in another independent cohort of fresh-frozen FL samples (*n* = 21 with progression <3 years vs. 20 indolent FL with progression >8 years; Fig. 6D) and lower *miR-29a* and *miR-29c* levels were associated with higher FL grade (Supplementary Fig. S30). The *miR-29s* expression did not predict OS or PFS in de novo DLBCL (*n* = 174; Supplementary Figs. S31, S32), which is in line with the known decrease of de novo DLBCL’s dependence on T-B-cell interactions compared to FL [48]. We also examined the association of a T-cell specific *miR-31* [44, 45, 49, 50] downmodulated in tFL (Fig. 1A) with FL prognosis revealing a robust association of its lower levels with shorter PFS in both discovery and validation cohort (data not shown). *miR-31* regulates the exhaustion of CD8+ cells, and low *miR-31* results in upregulated CD40L in Tfh cells [44, 45], but the mechanistic explanation of why its expression is prognostic in FL will require further detailed functional studies. Notably, for the miRNA quantifications in discovery and validation cohort, we isolated RNA from archival FFPE samples (stored at room temperature), which often led to RNA of low quality (median RIN = 1.75, range: 1.1–2.5), but this did not interfere with qRT-PCR quantification of miRNAs representing a significant advantage for their use as biomarkers. Overall, lower *miR-29s* expression and higher TRAF4 levels associated with increased FL aggressiveness, however, this is likely modified by other microenvironmental interactions and/or the context of genetic aberrations.

## Discussion

Here, we show that in tFL, MYC-mediated *miR-29* repression leads to subsequent upregulation of its target TRAF4 and increased CD40L signaling. The gain of CD40 activity can serve as an important driver of tFL activation and correlates with increased proliferation rate in FL/tFL. Notably, T-cell content is generally reduced in tFL, suggesting that this pathway represents a tFL cell adaptation to relatively lower access to CD40L + T-cells. Low *miR-29s* levels associated with unfavorable FL prognosis in a discovery and validation cohort from SWOG S0016 R-CHOP trial and can be reliably quantified from archival FFPE tissue.

We performed the first whole genome profiling of microRNAs (i.e. short non-coding RNAs) upon FL transformation (*n* = 10 FL-tFL pairs). This confirmed *miR-150* downmodulation in tFL [9] but also revealed changes in other miRNAs, including all members of the *miR-29* family. This caught our attention, since *miR-29s* have been implicated in multiple hematological malignancies including Burkitt lymphoma, mantle cell lymphoma, chronic lymphocytic leukemia, acute myeloid leukemia, and multiple myeloma, acting mostly as a tumor suppressors (reviewed in refs. [47, 51]) [19, 28–31, 52]. Consistent downmodulation of *miR-29s* in tFL led us to investigate their biological role. To identify the processes potentially regulated by *miR-29s* downmodulation in FL/tFL, we performed mRNA profiling of FL-tFL paired samples. To our knowledge, this is the first RNA profiling of paired FL-tFL samples that includes long (mRNAs) and short RNAs (miRNAs). The mRNA profiling identified CD40 signaling as clearly enriched in tFL samples compared to paired FL samples, and this was validated in other datasets [18]. Analyzing scRNA-seq data [26] revealed CD40-CD40L as one of the most important receptor-ligand pairs responsible for gene expression differences between FL and tFL. To uncover *miR-29* targets, we next generated two lymphoma B-cell lines engineered for *miR-29c* over-expression. This identified 20 mRNAs affected by *miR-29s* overexpression in both models, but also revealed cell line specific effects on transcriptome. The overlap of these data with the mRNA profiling from FL-tFL pairs revealed 7 anticorrelated mRNAs with putative *miR-29* binding sites. This included TRAF4, a crucial positive CD40 signaling regulator [29], and identified it as being repressed by *miR-29s* in vitro and upregulated in tFL in vivo. Importantly, functional studies showed that *miR-29* overexpression reduced TRAF4 levels and led to impaired B-cell lymphoma responsiveness to CD40 ligation (reflected in decreased NF-kB activity). On the other hand, over-expression of TRAF4 rescued the effects of *miR-29s* on CD40 signaling. These findings reveal for the first time the increase in CD40 signaling in the great majority of tFL (~90%) and the role of *miR-29* repression in this process.

It is well known that CD40 signaling is important pro-proliferative signal in normal B-cells, and in FL cells, but its increased activity in tFL is unexpected since aggressive lymphomas are generally considered to mostly lose dependence on microenvironmental interactions, including T-cell help [53–55]. The gain of CD40 pathway activity in tFL likely stems from CD40L’s key role in FL. CD40L on Tfh cells stimulates FL cells into proliferation and can protect them from apoptosis [53, 54]. Tfh cells positively select GC B-cells predominantly through CD40L engagement [55], MYC inductions, and synergistic BCR activation [29]. Most importantly, constitutive CD40L expression on T-cells directly promotes lymphomagenesis in mice [14, 56]. FL cells also frequently harbor HVEM (*TNFRSF14*) or *KMT2D* loss, which both lead to amplified CD40 signaling [41]. Here, we reveal that *miR-29* downmodulation represents a novel mechanism that tFL cells utilize to increase CD40 signaling propensity. These observations suggest that tFL cells retain some of the T-cell dependencies typical for FL cells. A recent spatial analysis of FL niches showed enrichment of Tfh and T regulatory cells and their physical proximity to FL B-cells, and ligand-receptor interactions [48]. Tfh cells are the main source of CD40 ligand, and their increased levels, activity and spatial organization were associated with poor FL prognosis [57, 58]. The T-cell composition in tFL has been less characterized, but differs from FL and de novo DLBCL [12]. Immunohistochemistry staining and in silico deconvolution of cell types from our bulk mRNA profiling of FL-tFL pairs revealed a significant reduction in CD4+ and CD8+T-cell content. This is in line with tFL cells engaging immune evasion and escaping contact with cytotoxic T-cells [4, 12, 13]. Interestingly, the content of Tfh cells, the major source of CD40L, did not seem to be reduced in tFL (CYBERSORTx). This is partially in line with Sarkozy et al., who found that in 4 of 11 paired FL–tFL biopsies analyzed by scRNA-seq, Tfh cell content was increased or unchanged post-transformation, while the rest showed reduced Tfh numbers [12]. On the other hand, tFLs have nearly universal reduction of T-central-memory cell fractions compared to paired FL, suggesting that tFL cells potentially tend to maintain Tfh interactions while reducing interactions with other T cell types [12]. Importantly, when we performed a ligand-receptor modeling (NicheNet tool) this showed that tFL cells interact with CD40L+ Tfh cells. In vitro increased TRAF4 levels helped to amplify CD40 signaling and proliferation. Indeed, higher TRAF4 protein levels were correlated with cell proliferation (Ki67) in lymphoma biopsies (IHC). This suggests that tFL cells amplify CD40 signaling propensity to potentially compensate for reduced CD40L availability caused by immune evasion that results in reduced T cell numbers. Overall, this is also in line with a mouse model in which constitutive CD40 activation leads to B-cell lymphoma development [14, 56], and this is further accelerated by concurrent depletion of lymphoma-limiting cytotoxic T-cells [59].

We further demonstrated that MYC is responsible for transcriptionally repressing *miR-29s* in tFL by binding to their promoters. MYC activity gain in tFL can be caused by its translocations [3, 4] but is often triggered by cell signaling aberrations [60] since most tFLs do not harbor MYC genomic aberrations to explain its activity in tFL[4, 5]. Indeed, MYC gain-of-function activity was present in all our tFL samples, irrespective of the presence of its translocation (3 out of 13 tFL). Considering that CD40 signaling is increased in tFL, we hypothesize that this could also contribute to MYC activity since CD40L is its canonical driver [61]. The FL-tFL transcriptomic data analysis for causal upstream networks indicated that CD40L acts as an MYC inducer in tFL via increased ERK activation (Supplementary Fig. S33), a well-known MYC inducer [62, 63]. This suggests a feed-forward loop where CD40L upregulates MYC, which further increases CD40 signaling propensity through *miR-29s* repression and TRAF4 upregulation. This resembles the role of *miR-150* repression via MYC in increasing BCR activity in CLL/tFL [9, 20, 23].

The *miR-29* family and their targets might also be relevant to the FL aggressiveness before transformation since it has been previously shown that *miR-29* levels are prognostically relevant in mantle cell lymphoma, acute myeloid leukemia and multiple solid tumors [30, 51, 64]. Indeed, low *miR-29a/b*/*c* levels associated with higher FL grade and with shorter OS and PFS in FL (*n* = 185), and *miR-29c* prognostic relevance was validated in an independent cohort (*n* = 92) from the R-CHOP clinical trial. In all *miR-29s* biomarker studies, we used RNA isolated from archival FFPE samples (often >15 years old), underscoring the feasibility of assessing miRNA-based biomarkers from low quality RNA. The prognostic performance of *miR-29c* was comparable to using complex genomics profiling [9, 65], including genomic characterization of the samples from the same SWOG S0016 cohort [25]. It would be interesting to test in future a combination of several miRNAs with prognostic relevance in FL to generate a miRNA-expression score that can be assessed from FFPE samples using an absolute miRNA copy quantification [20] to prospectively identify patients with unfavorable prognosis.

From a translational perspective, the reduced *miR-29* levels and increased CD40 signaling in tFL might be therapeutically targetable. The CD40 pathway has been explored clinically using both agonistic and antagonistic approaches in B-cell lymphomas [66–68]. Notably, one third of FL patients had a clinical response to lucatumumab, a fully humanized antagonistic anti-CD40 monoclonal antibody [67]. In contrast, no TRAF4 inhibitors or degraders are currently in development, although TRAF4 appears to be an attractive target across several cancer types [69, 70]. Importantly, *miR-29* mimic remlarsen (MRG-201) has been clinically tested in fibrotic disorders providing proof-of-concept data for such *miR-29*-based therapeutics (trial NCT03601052) [71]. Moreover, it has been shown that synthetic *miR-29* can be specifically delivered to malignant CLL cells via conjugation with antibodies against cell-surface molecule ROR1 [72].

**Fig. 7:**
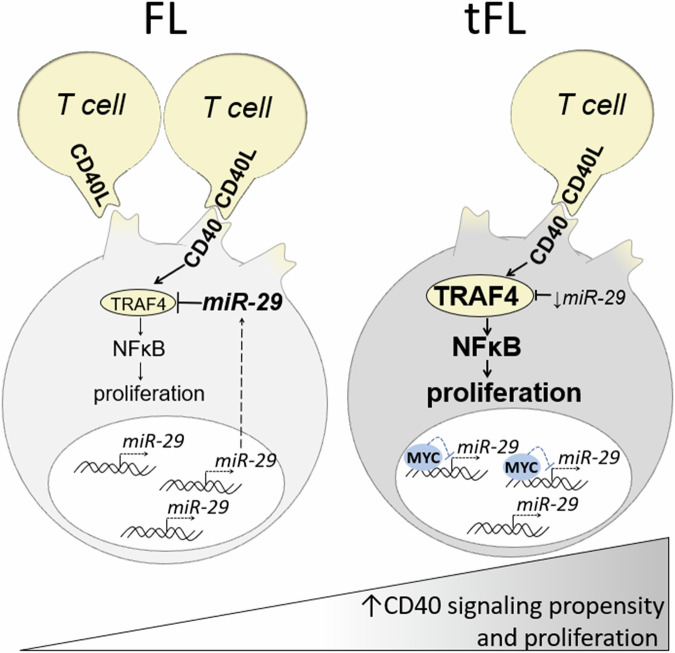
Schematic overview of *miR-29s* role in CD40-induced NF-kB signaling in tFL via regulation of TRAF4 expression. MYC activity gain upon FL transformation due to genetic aberrations or signaling activation downregulates *miR-29* expression in malignant B-cells. This leads to increased levels of its direct target TRAF4, which acts as a positive regulator of CD40 signaling. Consequentially, despite the decrease in numbers of T-cells upon FL transformation, this causes an increase in CD40 pathway activity, which leads to stronger NF-kB signaling.

In conclusion, we show that in tFL, MYC represses *miR-29s* levels, leading to an increase in TRAF4 levels and subsequently stronger CD40 signaling propensity and cell proliferation (summarized in Fig. 7). The low levels of *miR-29s* can be used as biomarkers of unfavorable FL prognosis accessible from FFPE tissue.

## Supplementary information

Filip et al manuscript major revisions SUPPLEMENT

## Data Availability

The miRNA/RNA-seq data have been deposited to EGA. Results of their analysis may be found in a data supplement. mRNA-seq: EGAD50000001384, https://ega-archive.org/datasets/EGAD50000001384. miRNA-seq: EGAD50000001385, https://ega-archive.org/datasets/EGAD50000001385.
