# Supplementary material for: Repression of miR-29 via MYC leads to increased CD40 signaling in transformed follicular lymphoma
Source: Leukemia. 2026 Feb 19;40(4):759–72. doi: 10.1038/s41375-026-02868-8 (PMC13056591; doi:10.1038/s41375-026-02868-8)
Supplement: Supplementary file 1 — Filip et al manuscript major revisions SUPPLEMENT [file 41375_2026_2868_MOESM1_ESM.pdf]

Supplemental Materials

Filip et al. 2026

Correspondence:

Marek Mraz, M.D., Ph.D.
Professor of Oncology
Central European Institute of Technology, Masaryk University
Kamenice 5, 625 00 Brno, Czech Republic
E-mail: marek.mraz@email.cz
Tel.: +420 549498143

1) Supplemental Methods

2) Supplemental Figures: 33

3) Supplemental Tables: 6

1) Supplemental Methods

Patient samples and cell lines

Biological material was obtained based on approval by Institutional Review Board, with written informed consent and according to the declaration of Helsinki. Only grade 1-3A was considered as follicular lymphoma in our study. Eleven pairs of clonally related FL-tFL samples (Formalin-fixed paraffin-embedded [FFPE]) were analyzed (n=22) by mRNA-seq and 10 FL-tFL pairs were analyzed by miRNA-seq (9 pairs overlapping with mRNA-seq) (supplemental Table 1). All the analyzed tFLs were clonally related and histologically verified transformations to DLBCL. For testing of miRNAs as biomarkers in FL we utilized a discovery cohort (n=185, FFPE samples), validation cohort from the R-CHOP arm of the S0016 trial (NCT00006721[25,26]; n=92 available FFPE samples), and DLBCL FFPE samples (n=174); cohorts characteristics in supplemental Table 2-4. Additional FL-tFL pairs (n=10 pairs), tFL (n=15), FL (n=94) and DLBCL (n=30) samples were used in additional analysis. SU-DHL4, KARPAS422, DOHH2, WSU-NHL, HEK293-FT, and HS5 cell lines were obtained from DSMZ and ATCC and cultured (5% CO_2, 37ºC) in recommended media with 10% FBS (Biosera) and 100 $U \cdot ml^{-1}$/100 $\mu g \cdot ml^{-1}$ penicillin/streptomycin (Sigma Aldrich). For functional studies and experimental procedures, see supplemental Methods.

RNA isolation and gene expression analyses by qRT-PCR

RNA from FFPE used for miRNA-seq was isolated from 10-20µm tissue sections using High Pure miRNA Isolation Kit (Roche, Catalog #: 05080576001) according to the manufacturer's protocol. RNA for mRNA-seq, miRNA qRT-PCR and mRNA qRT-PCR was isolated from 10-20 µm FFPE tissue sections using AllPrep DNA/RNA FFPE Kit (Qiagen, Catalog #: 80234) according to manufacturer's protocol with single adjustment in prolonging the tissue lysis step from 15 minutes to 120 minutes to increase the total yield. Total RNA from freshly-frozen tissue, native tissue or cell lines was isolated with TRI-Reagent (Sigma-Aldrich) as described previously.[1] For mRNA and miRNA expression analysis, cDNA was synthesized using High-Capacity cDNA Reverse Transcription Kit. The expression of individual miRNAs by qRT-PCR was assessed by TaqMan microRNA Assays (Thermo Fisher Scientific) and gene expression was detected using TaqMan Gene Expression Assays (Thermo Fisher Scientific), both used according to manufacturer's protocol. The obtained data from expression of *miR-29a* (Thermo Fisher Scientific, Catalog #: 4440887, Assay ID: 002112), *miR-29b* (Thermo Fisher Scientific, Catalog #: 4440887, Assay ID: 000413), *miR-29c* (Thermo Fisher Scientific, Catalog #: 4440887, Assay ID: 000587) were normalized to *RNU38B*

(Thermo Fisher Scientific, Catalog #: 4440888, Assay ID: 001004) except for samples from SWOG cohort where we utilized geometric mean of *RNU38B*, *RNU6B,* and *miR-16* to increase normalization accuracy (Thermo Fisher Scientific, Catalog #: 4440888, Assay ID: 001006; Catalog #: 4440887, Assay ID: 000391). To perform qRT-PCR for mRNAs the following probes were used: *MYC* (Thermo Fisher Scientific, Catalog #: 4331182, Assay ID: Hs00153408_m1), *TRAF4* (Thermo Fisher Scientific, Catalog #: 4331182, Assay ID: Hs01030624_m1), *TBP* (Thermo Fisher Scientific, Catalog #: 4333769F).

miRNA and mRNA profiling by NGS

For miRNA profiling, 20 FFPE samples (10 pairs; for sample characteristics see supplemental Table 1) were used for library preparation with NEBNext Multiplex Small RNA Library Prep Set for Illumina (NEB,Catalog number: E7560S) according to manufacturer's protocol. Briefly, 800 ng of RNA per sample was used as input for library preparation and the prepared libraries were sequenced by NextSeq Sequencing System (Illumina). The data were processed as follows: Quality of the raw sequencing reads was checked using FastQC (v0.11.3).[2] Cutadapt (v1.15)[3] was used to remove the adapter sequence. The pre-processed reads were mapped and counted using Miraligner.[4] Differential miRNA expression was calculated by DESeq2.[5] Pairing of the samples was considered during differential gene expression calculation. Only miRNAs with a mean base count of >200 reads per million were kept for the final analysis. Mature and hairpin miRNA mappings/calculations were processed separately, and only mature miRNA results were considered.

For mRNA profiling, 22 FFPE samples (11 pairs; for sample characteristics see supplemental Table 1) were used for library preparation with QuantSeq 3' mRNA-Seq Library Prep Kit FWD (Lexogen, Catalog number: 015) according to manufacturer's protocol (without the modifications for FFPE samples). The amplification step of library preparation was performed as qRT-PCR by adding 1 ul of EVAGREEN dye (Biotium) to the reaction which allowed to determine correct amount of amplification cycles to avoid under or overamplification. We used 500 ng of total RNA as input and used Lexogen i5 6 nt Unique Dual Indexing Add-on Kit together with UMI Second Strand Synthesis Module for QuantSeq FWD during the library preparation according to protocol. Bcl files were converted to Fastq format using bcl2fastq v. 2.20.0.422 Illumina software for basecalling. Quality check of raw single-end fastq reads was carried out by FastQC.[6] The adapters and quality trimming of raw fastq reads was performed using Trimmomatic v0.39[7] with settings CROP:250 LEADING:3 TRAILING:3 SLIDINGWINDOW:4:5 MINLEN:35. Technical duplicates were identified based on their UMI and removed using UMI-tools.[8] Trimmed RNA-Seq reads were mapped against the human genome (hs38) and Ensembl GRCh38-p10 annotation using STAR v2.7.3a[9] as splice-aware short read aligner and default parameters except --outFilterMismatchNoverLmax 0.5 and --twopassMode Basic. We used 0.5 as threshold for outFilterMismatchNoverLmax parameter rather than default 0.66 due to the fact that reads produced by QuantSeq contain sections with poly-A due to the design of the primers in the kit. Quality control after alignment concerning the number and percentage of uniquely- and multi-mapped reads, rRNA contamination, mapped regions, read coverage distribution, strand specificity, gene biotypes and PCR duplication was performed using several tools namely RSeQC v4.0.0[10], Picard toolkit v2.25.6, and Qualimap v.2.2.2.[11] The differential gene expression analysis was calculated based on the gene counts produced using featureCounts from Subread package v2.0[12] and further analyzed by Bioconductor package DESeq2 v1.34.0.[5] Data generated by DESeq2 with independent filtering were selected for the differential gene expression analysis due to its conservative features and to avoid potential false positive results.

NGS data has been deposited at the European Genome-phenome Archive (EGA), which is hosted by the EBI and the CRG, under accession numbers EGAD50000001384 (mRNA-seq) and EGAD50000001385 (miRNA-seq).

Native tissue processing and cell sorting

Native lymphatic tissue was homogenized immediately after biopsy using cell dissociation sieve (Sigma-Aldrich). Cells were stained with anti-CD19 (Immunotech), anti-CD3 (Invitrogen), anti-CD4 (Invitrogen), anti-CD25 (B-D Biosciences), anti-CD8 (eBioscience) antibodies and sorted using FACS Aria III cell sorter (B-D Biosciences).

Cell transfection

The SU-DHL4 and WSU-NHL cells were electroporated using the Neon Transfection System (Thermo Fisher Scientific). SU-DHL4 cell line was electroporated with an synthetic *miR-29c* (MISSION miRNA Mimic, 1000 nM, Sigma Aldrich) or control short RNA (MISSION miRNA Mimic Negative Control, 1000 nM, Sigma Aldrich) and siRNA against *TRAF4* (Silencer Select Pre-Designed siRNA, 500 nM, Thermo Fisher Scientific) or control siRNA (Silencer Select Negative Control No.1, 500 nM, Thermo Fisher Scientific). For SU-DHL4 cell line, we used 2 pulses of 1200V for 20ms to transfect the cells and transfected cells were harvested after 48h for viability analysis and to be stimulated by CD40L. WSU-NHL cell line was transfected with siRNA against *MYC* (Silencer Select Pre-Designed siRNA, 500 nM, Thermo Fisher Scientific) or control siRNA (On-Target Plus Smartpool Negative, 1000

nM, GE Dharmacon). For WSU-NHL cell line, we used 1 pulse of 1500V for 20ms to transfect the cells and transfected cells were harvested after 48h for subsequent analysis. Primary cells from FL lymph nodes were transfected with synthetic *miR-29c* (mirVana miRNA mimic *hsa-miR-29c-3p*, 1000 nM, Catalog #: 4464066, Thermo Fisher Scientific) or control short RNA (mirVana miRNA negative control #1, 1000 nM, Catalog #: 4464058, Thermo Fisher Scientific) using Super Electroporator NEPA21 Type II (NEPA GENE) and the following poring pulse settings (300V, 1ms length, 50ms interval, 4 pulses, 10% D. rate, + polarity) and transfer pulse settings (20V, 50ms length, 50ms interval, 5 pulses, 40% D. rate, +/- polarity). The transfected cells were subsequently co-cultured with HS5-CD40L-IL4-IL21 cells (see below).

Cell viability analysis

Cell viability was investigated by DiOC6 (3,3′-dihexyloxacarbocyanine iodide) together with PI (propidium iodide) staining (Thermo Fisher Scientific).[13] All measurements were performed on FACS Verse (BD Biosciences-US).

CD40L stimulation and HS5/HS5-CD40L-IL4/IL21 cocultures

Cell lines were stimulated with recombinant soluble CD40 ligand (CD40L, 1 mg/mL; Peprotech) in serum-free media (37°C) for 3/5/10 min and placed on ice immediately after stimulation. Cells were then lysed for immunoblotting. For co-culture experiments we utilized engineered HS5 cell line created as described in Hoferkova et al., 2024.[14] HS5-CD40L or HS5-5 wildtype (non-irradiated) cells were seeded at 1×10^6 in 6-well plate in DMEM with 10% FBS. The next day, DMEM medium was replaced by RPMI media containing 10% FBS and 6×10^6/ml of SUDHL4 cells. The coculture was conducted for 6h and cells were then harvested. Cells were stained with Sytox blue (Thermo Fisher), anti-CD105 antibody (Sony) and anti-CD19 antibody (Sony) to validate purity of B cells that were harvested.

For experiments with primary FL cells, transfected cells from FL lymph nodes ($2x10^6$ cells per condition) were cocultured with HS5-CD40L-IL4-IL21 cells ($0.5x10^6$ cells per condition, non-irradiated) for 96 h. All cells in the well were harvested and stained using eFluor 450 fixable viability dye, fixed with 4% PFA, permeabilized using 0.01% TX-100, blocked by 3% BSA with 0.05% Tween 20 in PBS and stained over night with anti-Ki67 primary antibody (Cell Signaling, D3B5 Rabbit mAb #9129). Next day fixed cells were stained with a secondary anti-rabbit antibody (Cell Signaling, Anti-rabbit IgG Alexa Fluor 647 #4414), anti-CD19 antibody (Sony) and anti-CD105 antibody (Sony).

Preparation of *miR-29c* overexpressing

Plasmid pLKO.1 puro (Addgene, #8453) was used as backbone for preparing plasmid for *miR-29c* overexpression. Oligo containing sequence for *pre-miR-29c* as well as restriction sites for EcoRI (NEB) and MIuI (NEB) restriction enzymes was ordered from Sigma-Aldrich.

5′CCGGTGACCGATTTCTCCTGGTGTTCAGAGTCTGTTTTTGTCTAGCACCATTTGAAATCGGTTATTTTTG 3′
3′____ACTGGCTAAAGAGGACCACAAGTCTCAGACAAAAACAGATCGTGGTAAACTTTAGCCAATAAAAACTTAA5′

Proper integration of insert into plasmid was always validated by Sanger sequencing. The envelope plasmid CMV-VSV-G (Addgene, #8454) was purchased from Addgene while packaging plasmid dR8.91 was kindly provided by Dr. M. Smida (Masaryk University). Virus particles were produced in HEK-293-FT (obtained from ATCC) after plasmids transfection by PEI (Polysciences) and added to desired B cell lines. B cell lines transduced with *miR-29c* overexpressing plasmids were subsequently selected by puromycine (10 µg/ml, Sigma Aldrich) for 5 days.

Preparation of TRAF4 overexpressing cell lines through viral transduction

For TRAF4 overexpression, we designed primers using Takara Primer design tool andthe following primers were ordered from Sigma-Aldrich.

TRAF4 FWD: GGAGAATTGGCTAGCGAATTGCCACCATGCCTGGCTTCGACTACAAG

TRAF4 REV: GTTCGTGGCTCCGGAACCGGTCAGCTGAGGATCTTCCGGG

The TRAF4 sequence (without its 3'UTR) was cloned into pCW57-MCS1-P2A-MCS2 plasmid pCW57-MCS1-P2A-MCS2 (Addgene, #234619). To obtain TRAF4 sequence, RNA was isolated from blood of healthy donor and used to synthesize cDNA (Transcriptor High Fidelity cDNA Synthesis Kit, Roche). Amplification of TRAF4 was performed by PCR using designed primers and Q5 high-fidelity DNA polymerase (Q5 High-Fidelity DNA Polymerase, NEB). Final size of the amplified product was confirmed by electrophoresis on agarose gel and the product was isolated from agarose gel using a gel extraction kit (QIAquick Gel Extraction Kit, QIAGEN). Plasmid was linearized by AgeI-HG (NEB) and EcoRI-HG (NEB). TRAF4 was inserted into linearized plasmid using In-Fusion HD EcoDry Cloning Kit (Takara). Proper integration of insert into plasmid was validated by Sanger sequencing. The envelope plasmid CMV-VSV-G (Addgene, #8454) was purchased from Addgene while packaging plasmid dR8.91 was kindly provided by Dr. M. Smida (Masaryk University). Virus particles were produced in HEK-293-FT (obtained from ATCC) after plasmid transfection by PEI (Polysciences). DOHH2 cell line transduced with

virus particles encoding TRAF4 were selected by blasticidine (10 µg/ml, Thermo Fisher) for 5 days. For the rescue experiment, overexpression of TRAF4 was induced by doxycycline (1 µg/mL, Sigma Aldrich) for 24hrs, followed by DOHH2 cells being transfected with synthetic *miR-29c mimic* or control miRNA mimic as described above. After transfection, cells were continuously cultured in the presence of doxycycline (1 µg/mL, Sigma Aldrich) for another 24hrs and then stimulated with CD40L for 10 min as described above.

Immunoblotting

Cells were lysed in lysis buffer (1% SDS, 50 mM TRIS-HC pH 6.8, 10% glycerol) with phosphatase and protease inhibitors (Sigma Aldrich) and protein concentration was determined using DC Protein Assay (BioRad). Equal amounts of protein were separated by SDS-PAGE and transferred to the PVDF membrane (0.45 µm pore size, Millipore). The membranes were incubated with the Cell Signaling antibodies specific for the following immobilized proteins: TRAF4 (#18527, 1:1000), pIKK alpha beta (#2697, 1:1000), IKK alpha (#61294, 1:1000), IKK beta (#2684, 1:1000), GAPDH (#2118, 1:1000) or Santa-Cruz antibody specific for Vinculin (sc-73614, 1:1000). Secondary horse-radish peroxidase (HRP)-conjugated anti-mouse or anti-rabbit antibodies from Cell Signaling (anti-rabbit #7074, anti-mouse #7076) were used to detect primary antibodies. Immunocomplexes were detected using ECL (BioRad), and the chemiluminescent signal was digitally detected with UVItec Alliance 4.7 (UVItec). Densitometric quantification of immunoblots was performed using ImageJ Fiji. TRAF4 and pIKKαβ quantification was normalized to quantification of loading control.

Immunohistochemistry

The FFPE tissue sections (1 µm) were automatically processed and stained in VENTANA BenchMark Ultra system. Universal DAB Detection Kit (Ventana) was used for staining in combination with UltraView Amplification Kit (Ventana). Counterstaining was achieved by using Hematoxylin II (Ventana) and post-counterstaining by Bluing reagent (Ventana). Immunostaining was performed using the anti-TRAF4 antibody (D1N3A clone, 1:50; Cells Signalling), anti-MYC antibody (Y69 clone, 1:300; Abcam), anti-CD4 (SP35 clone,1:200; Roche), anti-CD8(C8/144B clone, 1:100; Dako) and anti-Ki67 (MIB-1 clone,1:200; Zeta Corp). Staining quantification was performed either using H-DAB color deconvolution by ImageJ Fiji or percentage of positive cells was determined by a trained pathologist. Normalized staining intensity was calculated as log10 of max value/mean value from measurement of deconvoluted picture.

CHIP-PCR

Briefly, $20x10^6$ SU-DHL4 cells were fixed in 1% formaldehyde (Thermo Fisher Scientific). The fixation was quenched by glycine after 10 min. After washing the fixed cells with cold PBS, pellet was snap-frozen in liquid nitrogen. In the next step, the cells were lysed in 300µl of Breaking Buffer (50 mM Tris-HCl pH 8.0, 1 mM EDTA, 150 mM NaCl, 1% SDS, 2% Triton X-100) complemented with protease/phosphatase inhibitors (1:100). Samples were sonicated on ice (UP100H sonicator, Hielscher, fitted with an MS1 sonotrode), which consisted of 18 cycles of 10-second pulses at 80% amplitude, each followed by a 1-minute incubation on ice. Cell debris was removed by centrifugation, and the supernatant was adjusted to a final volume of 2 ml with Triton buffer (50 mM Tris-HCl, pH 8.0; 1 mM EDTA; 150 mM NaCl; 0.1% Triton X-100). Chromatin was precleared with 50 µl of protein A/G ChIP-grade magnetic beads (Thermo Fisher Scientific) for 1 h. The beads were then discarded, and 5 µg of rabbit anti-c-Myc antibody [Y69] (ChIP grade; Abcam) or 5 µg of control rabbit IgG (12-370; Merck Millipore) was added per 1 ml of chromatin. The samples were rotated and incubated at 4°C overnight. The next day, the immunocomplexes were pulled down by 50 µl of A/G ChIP-grade magnetic beads (Thermo Fisher Scientific). Beads were washed sequentially in 500 µl each of Low Salt Immune Complex Wash Buffer (20-154; Merck Millipore), High Salt Immune Complex Wash Buffer (20-155; Merck Millipore), and LiCl Immune Complex Wash Buffer (20-156; Merck Millipore), and finally twice in 500 µl of TE buffer (50 mM Tris-HCl, pH 8.0; 1 mM EDTA). The elution was performed in 100 µl of Elution buffer (50 mM Tris-HCl pH 7.5, 5 mM EDTA, 1% SDS, 10 mM DTT). Samples were incubated for 4 hrs at 55°C with RNase A and Proteinase K. The DNA was purified by Zymo ChIP DNA Clean & Concentrator kit (#D5205; Zymo Research) and eluted in 30 µl of nuclease-free water (pre-warmed to 50°C). qPCR was performed using PowerTrack SYBR Green Master Mix for qPCR (Thermo Fisher). A melting curve analysis was performed to ensure that no primer–dimers were formed, and the product size was verified by agarose gel electrophoresis. The following primer sequences were obtained from Chang et al., 2008.[15]

miR-29a/ miR-29b-1 C1:
F- CACCAACTGAAAACCTGCCA
R- GAATGAACGTTGTGAAATCCCTC

miR-29a/ miR-29b-1 C2:

F- TGCGCGTGACCAGAAAAGTA
R- GCCTCAGATTGGTTCGCTTG

miR-29b-2/ miR-29c C3:
F- AGGGAGCCAACATGGAGACA
R- CGTTGGAAAGTTGTTTACCTTGC

Statistical analysis
Differences in expression levels between paired samples were compared using paired t-test or Wilcoxon matched pairs test and differences in non-matched groups were compared using unpaired t-test or Mann-Whitney U test. The appropriate tests were chosen based on the results of Shapiro-Wilk test of normality of input data. Overall survival (OS) was defined as the time between the analyzed biopsy and death and progression-free survival (PFS) as time between biopsy and first progression from biopsy. Survival curves were estimated using Kaplan-Meier method and differences in OS or PFS were compared using log-rank test. Statistical analyses for OS and PFS were performed using GraphPad Prism Software v8.0.1 (GraphPad Software, Inc.). The effect of various risk factors on PFS/OS was analyzed using univariate and multivariate Cox proportional hazard regression analyses using Statistica 13.2 (TIBCO Software Inc.). The left truncated Cox proportional hazards models (delayed entry models) were used for OS as the time of biopsy from diagnosis varied among patients. The left truncated Cox proportional hazards models (delayed entry models) for PFS were calculated from the initiation of therapy. For univariate analysis, miRNAs were dichotomized as low or high using the ≤median vs. > median, 1st tercile vs. tercile 2-3, and 1st quartile vs. quartile 2-4 of its relative expression. For multivariate analysis, the miRNA dichotomization with the lowest P value from univariate analysis was used. The multivariate analysis used Breslow likelihood with a stepwise Backward removal of P values >0.15 All statistical tests were 2-sided and P-values <0.05 were considered significant.

Computational analyses
ImageJ2 FIJI (v2.9.0/1.53t) was used for IHC analysis.[16] Gene set enrichment analysis (GSEA) was done using GSEA app (v4.1.0).[17,18] Counts normalized by DESeq2 were used as input for GSEA analysis. Our mRNA-seq data were also analyzed with the use of QIAGEN IPA (QIAGEN Inc., https://digitalinsights.qiagen.com/IPA). We performed standard expression analysis in IPA app using adjusted P value, base mean and log2 fold change from DESeq2 as input. For data from Parsa et al., 2020[19], average expression, logFC and adjusted P value from limma were utilized. Fold change was utilized to calculate z-score for IPA analysis. We focused on Upstream Analysis section and Pathways section. We used package Seurat (v4.3.0)[20] that allowed us to process and visualize scRNA-seq data from Roider et al., 2019.[21] We used package VIPER (v1.32.0)[22] with regulon from package bcellViper (v1.34.0) to assess TF activity in our mRNA-seq data. We performed multi-sample analysis (msVIPER) according to user manual provided by the authors and utilized normalized counts from DESeq2 as input. For analysis of data from Parsa et al., 2020[19] we utilized counts normalized by limma. Next, we utilized package decoupleR (v2.4.0)[23] in combination with gene regulatory network DoRothEA (v1.10.0)[24] to infer TF activity from RNA-seq data. We also utilized decoupleR with package PROGENy (v1.20.0)[25] to infer pathway activity from RNA-seq data. Analysis performed by decoupleR with DoRothEA or PROGENy were performed according to manual kindly provided by authors. Analysis by decoupleR is performed using stat parameter calculated by DESeq2. For data from Parsa et al., 2020[19], decoupleR analysis is performed using t value calculated by limma. For data from Roider et al.,2019[21], normalized gene expression was used as input for decoupleR analysis with DoRothEA. Package nichenetr (v1.1.1) was used for analysis of cell interaction in scRNA-seq data from Roider et al.,2019 using tool NicheNet[26] according to user guide kindly provided by authors. The whole Seurat object was used as input for NicheNet analysis including gene expression and cell type annotation. CARNIVAL package (v2.8.0)[27] was utilized according to authors user guide together with both DoRothEA and PROGENy outputs from our FL-tFL mRNAseq analysis as inputs for CARNIVAL. OmnipathR package (v3.9.3)[28] was used as prior knowledge network and IBM ILOG CPLEX Optimization Studio 22.1.1 obtained under academic license were used as ILP solver in CARNIVAL analysis. CIBERSORTx[29,30] online tool was employed for estimation of the abundances of cell types in a mixed cell population, using bulk gene expression data. Counts normalized by DESeq2 were used as input for CIBERSORTx analysis. Heatmaps in the paper were generated using packages gplots (v3.1.3) and ComplexHeatmap (v2.14.0). T-cell specific gene markers were derived from reference [31,32].

2) Supplemental Figures

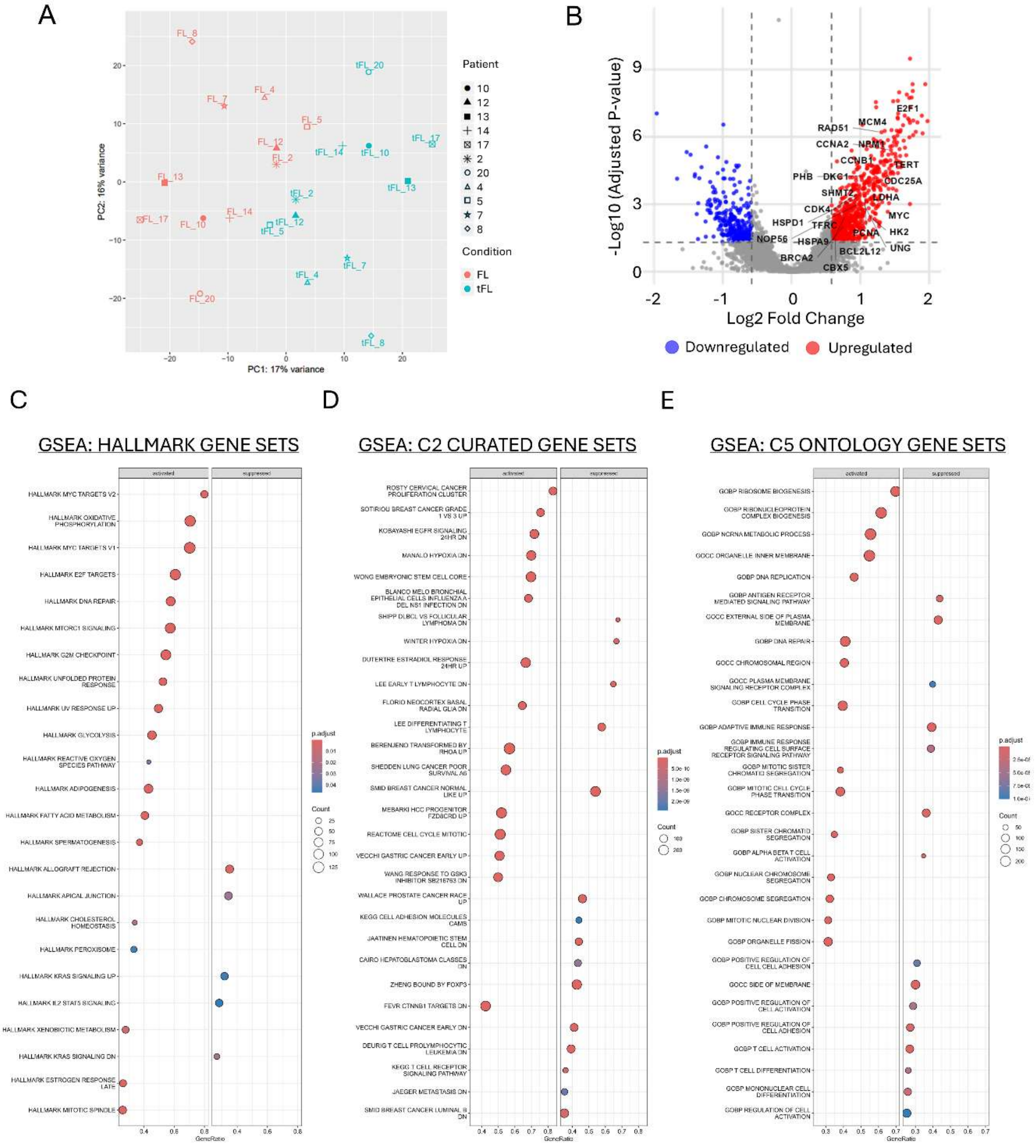

Figure S1: (A) Volcano plot depicting differentially expressed mRNAs (n=1,075, Padj<0.05, fold-change >1.5) in paired FL-tFL samples (n=11 pairs). Significantly downregulated mRNAs are indicated in blue, significantly upregulated in red and non-significant in grey. *MYC* and its targets are named. All tFLs were histologically verified as DLBCL. All RNA samples in the analysis were isolated from FFPE tissue. **GSEA enrichment analysis performed on data from mRNA-seq of paired FL-tFL samples (n=11 pairs; RNA-seq in this study) using (B) Principal component analysis (PCA) of RNA-seq profiling in paired FL-tFL samples (n=11 pairs).** Each symbol represents one patient, color represents FL (red) or tFL sample (blue). **(C) hallmark signature database, (D) C2 curated genes sets database and (E) C5 gene ontology database.**

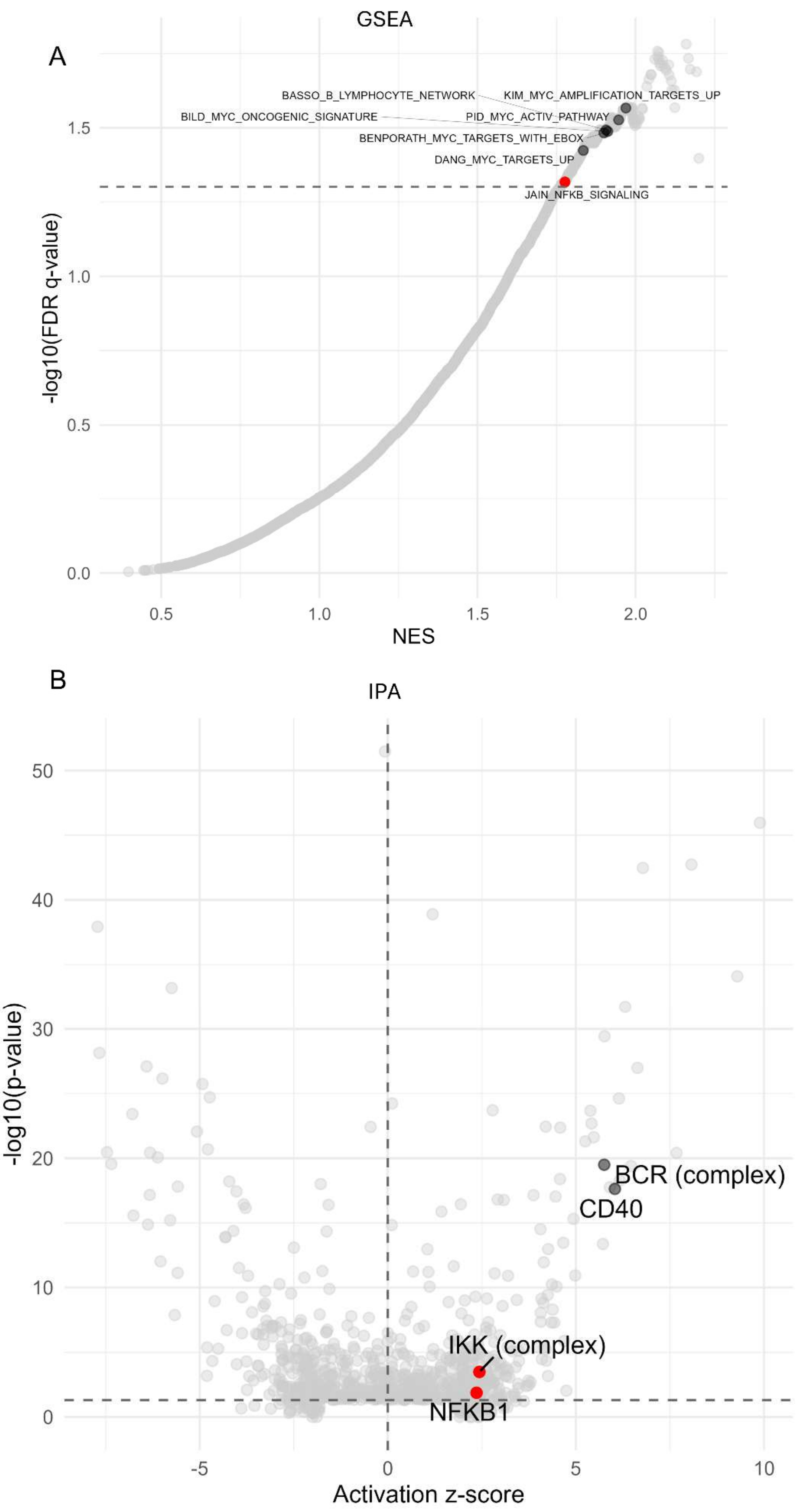

Figure S2: (A) GSEA analysis highlighting enrichment of NF-kB pathway (JAIN_NFKB_SIGNALING, NES=1.78, P=0.004) and other gene sets in curated gene sets database (C2) according to mRNA-seq of paired FL-tFL samples (n=11 pairs; RNA-seq in this study). (B) IPA analysis highlighting changes in the enrichment of NF-kB activity (IKK complex: z-score=2,44, P=0.0006; NFKB1: z-score=2.44, P=0.03) and others identified in mRNA-seq of paired FL-tFL samples (n=11 pairs; RNA-seq in this study).

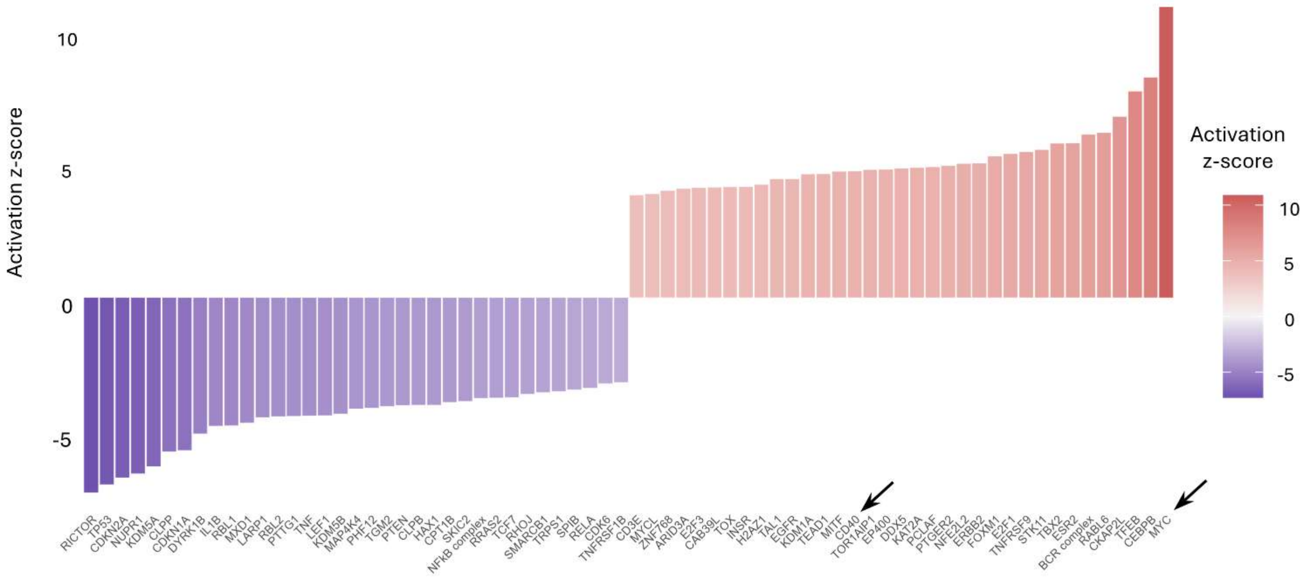

Figure S3: IPA tool enrichment analysis of upstream regulators using mRNA-seq data from fresh frozen FL-tFL samples (data from Parsa et al.[19], n=6 pairs). Red color indicates an increase in upstream regulator/pathway activity, and blue represents a decrease.

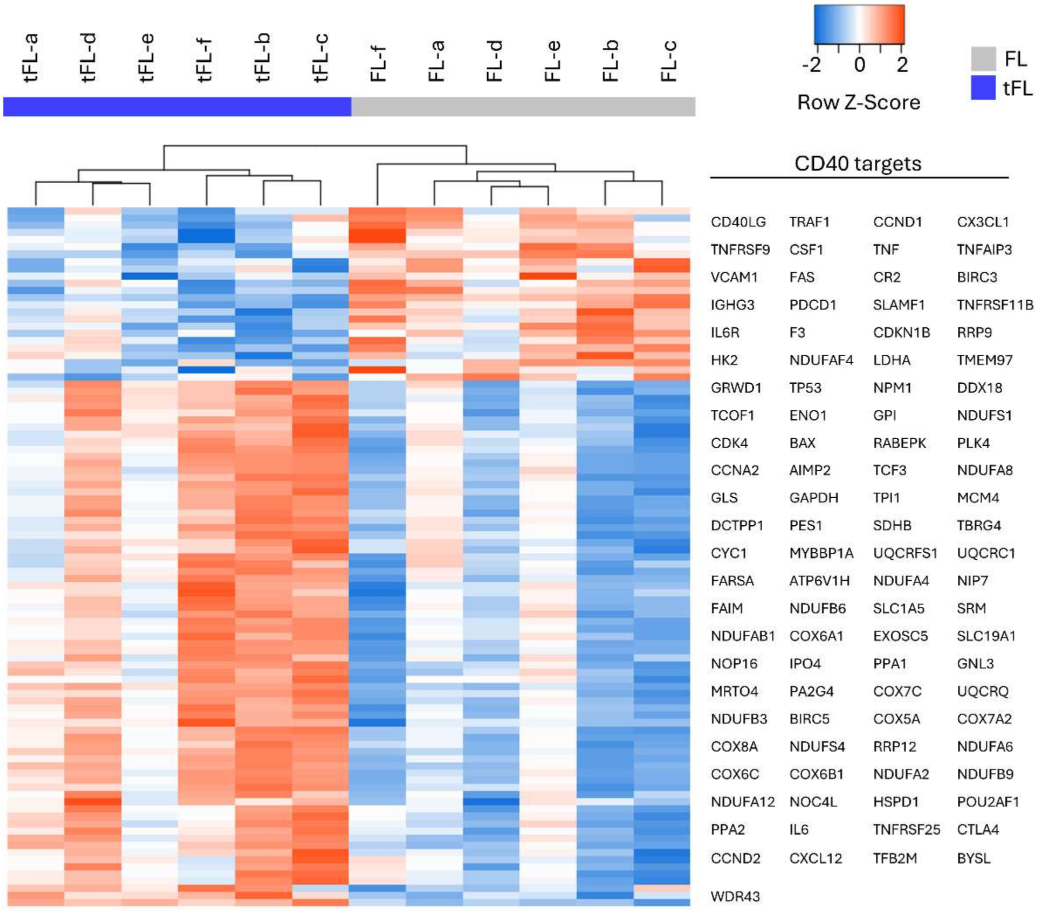

Figure S4: Heatmap representing expression of CD40 pathway targets (n=98) identified by IPA using mRNA-seq data from fresh frozen FL-tFL samples (data from Parsa et al.[19], n=6 pairs). Row z-score from normalized counts for each mRNA was plotted using online tool Heatmapper[33] (average linkage clustering method, Kendall's Tau distance measurement method). Lower expression indicated in blue, and higher expression in red.

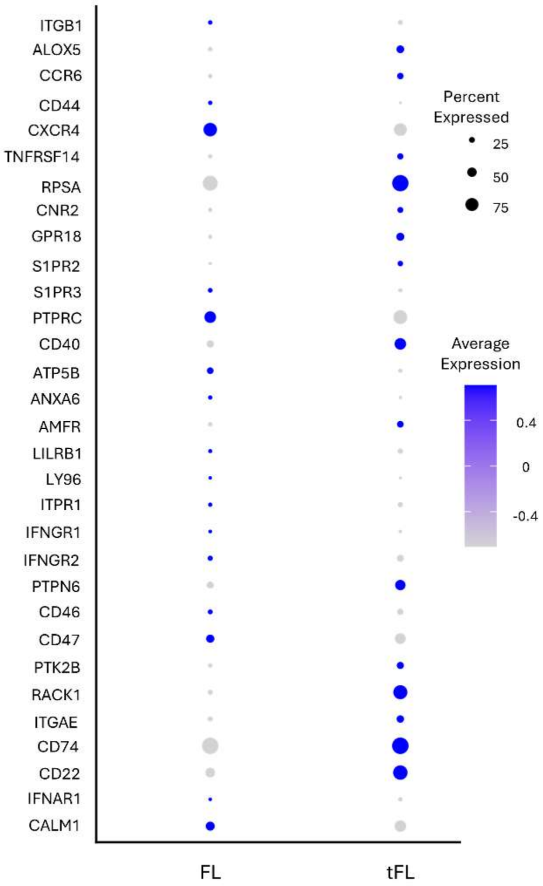

Figure S5: Expression visualization of FL cells receptors identified in NicheNet analysis between FL (n=4) and tFL (n=2) samples from Roider et al.[21].

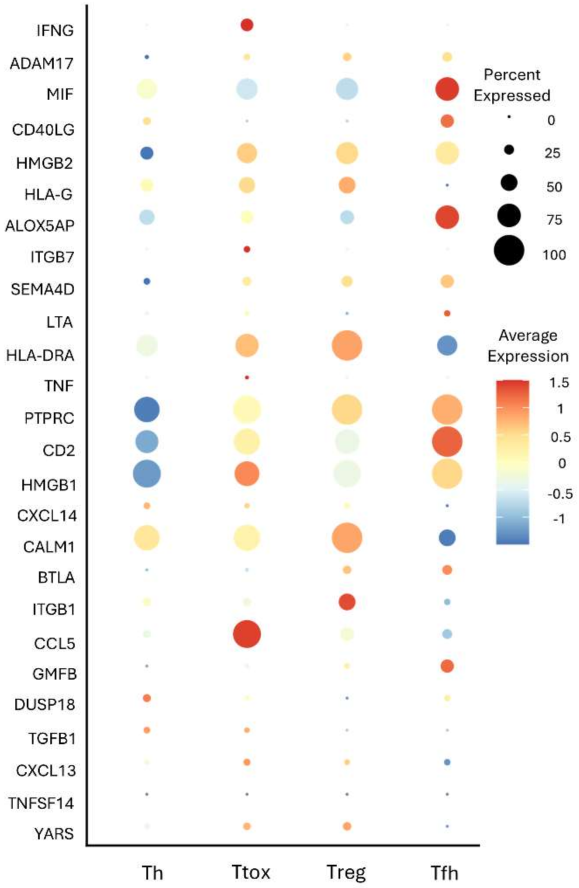

Figure S6: Difference in expression of ligands (NicheNet tool) between different T-cell types (TREG- T regulatory, TTOX- T cytotoxic, TH-T helper, TFH-T follicular helper) in Roider et al.[21].

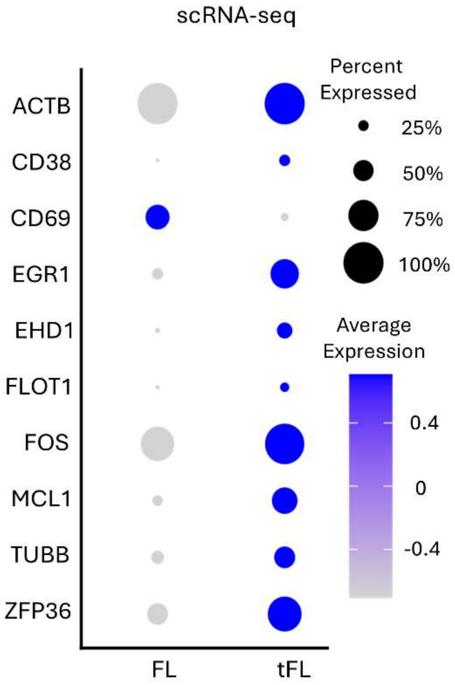

Figure S7: Expression comparison of genes regulated by CD40L (NicheNet analysis from scRNA-seq data) between FL and tFL samples (data from Roider et al.[21]).

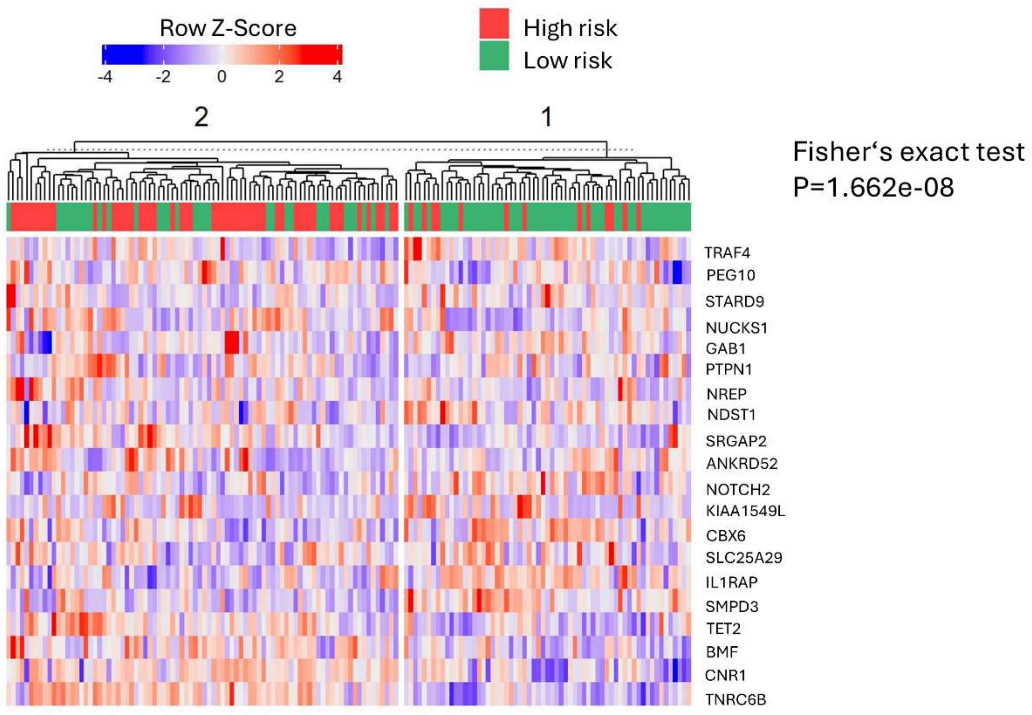

Figure S8: Heatmap visualizing separation of patients from Huet el al.[34] with relatively good vs. worse prognosis by expression of 20 *miR-29* targets. Expression of twenty *miR-29* targets (targets shared between SU-DHL4 and KARPAS422 cell lines over-expressing *miR-29c*) was used to cluster patients with low risk vs. high risk of progression (as defined in Huet et al.[34] according to PFS). The assignment of patients to good vs. bad prognosis was done based on the original data (Huet et al.[34]) using a panel of 395 mRNAs characterized in Huet et al.[34]. Clustering used 1000 iterations of k-means (ComplexHeatmap package). Fisher's exact test for enrichment of patients with aggressive disease in cluster 2 vs 1 ($P=1.662^{-08}$). Row z-score from normalized counts for each mRNA was plotted.

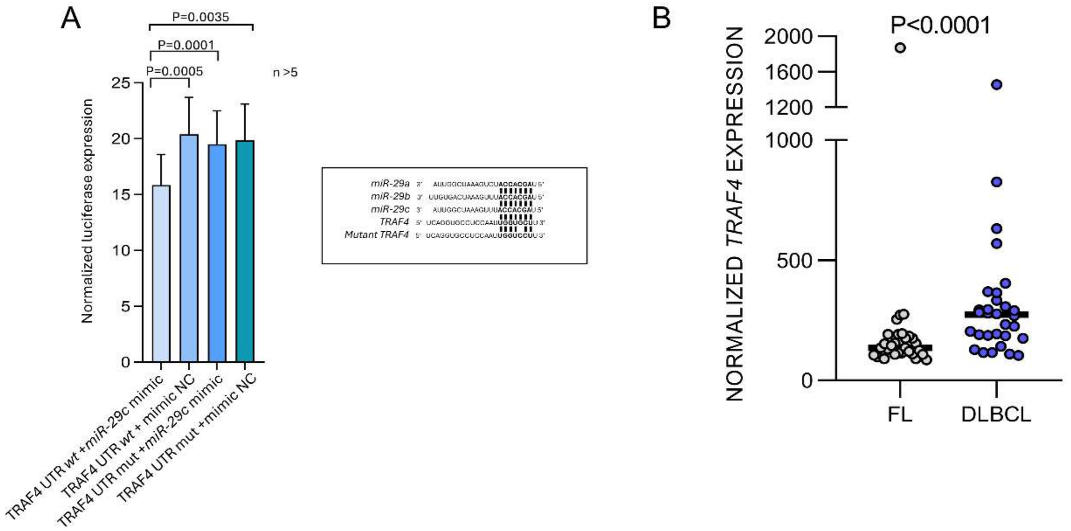

Figure S9: A) Luciferase activity in HEK293FT-cells co-transfected with psiCHECK2 vector containing the cloned 3'UTR region of *TRAF4* encoding the putative *miR-29* binding site at position 161-167 (TRAF4 UTR wt) and either synthetic *miR-29c* (*miR-29c* mimic) or control miRNA (mimic NC), or containing a cloned mutated 3'UTR of TRAF4 (TRAF4 UTR mut; (G to C at position 165) and either synthetic *miR-29c* (*miR-29c* mimic) or control miRNA (mimic NC). Renilla activity was measured 24 hours after transfection, and activity was normalized to the endogenous firefly control of the psiCHECK2 vector (n>5). The differences were compared by paired t-test. The error bars indicate SEM. Data obtained and reused from Sharma et al.[35]. **B)** Expression of *TRAF4* mRNA (qRT-PCR) in FL and *de novo* DLBCL (n=30 each). Statistical differences were compared by Mann-Whitney test.

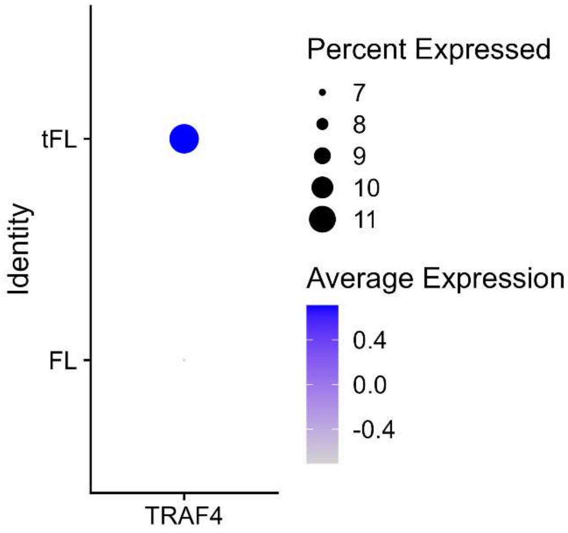

Figure S10: Difference in expression of *TRAF4* in malignant B cells between FL and tFL in Roider et al.[21]**.**

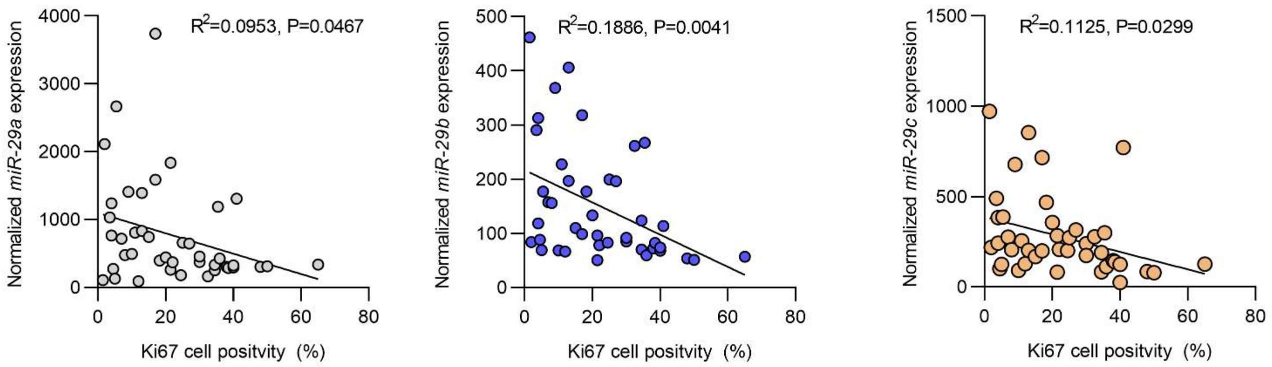

Figure S11: Significant anti-correlation of Ki67 staining (IHC) and *miR-29a/b/c* expression (n=43, qRT-PCR) in FL samples. Correlation calculated by Pearson correlation.

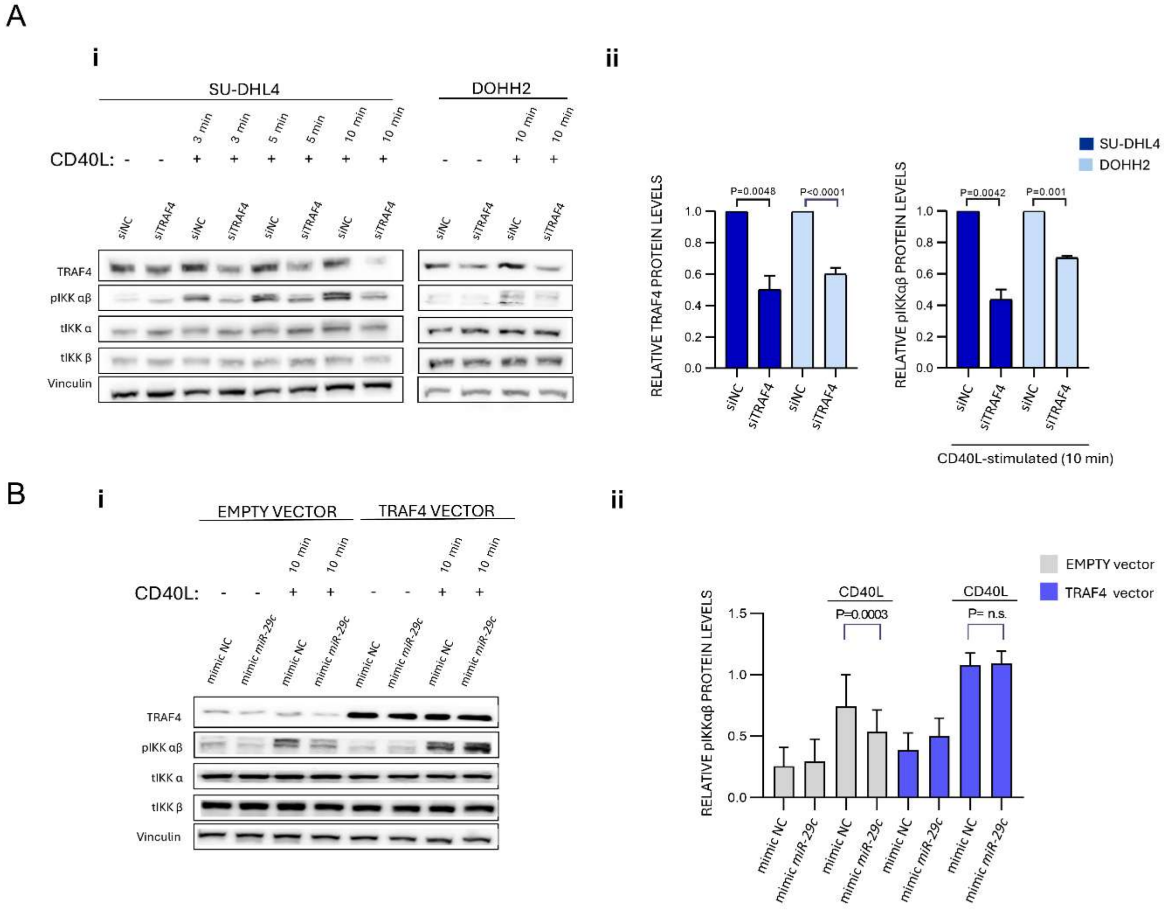

Figure S12: A) (i) Representative immunoblot analysis of SU-DHL4 and DOHH2 cells transfected with siRNA negative control (siNC) or siRNA against *TRAF4* (siTRAF4). Transfected cells were stimulated with CD40L (1 ug/ml, 3-10 min.). Vinculin was used as a loading control. **(ii)** Densitometric quantification of TRAF4 levels (siTRAF4 n=5) and pIKK levels in CD40L-stimulated samples (siTRAF4 n=3). **B) (i) Representative immunoblot from DOHH2 cell transduced with plasmid encoding TRAF4 (TRAF4 VECTOR without *TRAF4* 3'UTR) or control plasmid (EMPTY VECTOR), and subsequently transiently transfected with synthetic *miR-29c* mimic (mimic miR-29c) or negative control (mimic NC).** Transfected cells were cultured for 24 hrs and subsequently stimulated with CD40L (1 ug/ml, 10 min.) or vehicle. **(ii)** Densitometric quantification of pIKK levels in 3 replicates of the experiment described in [Bi].

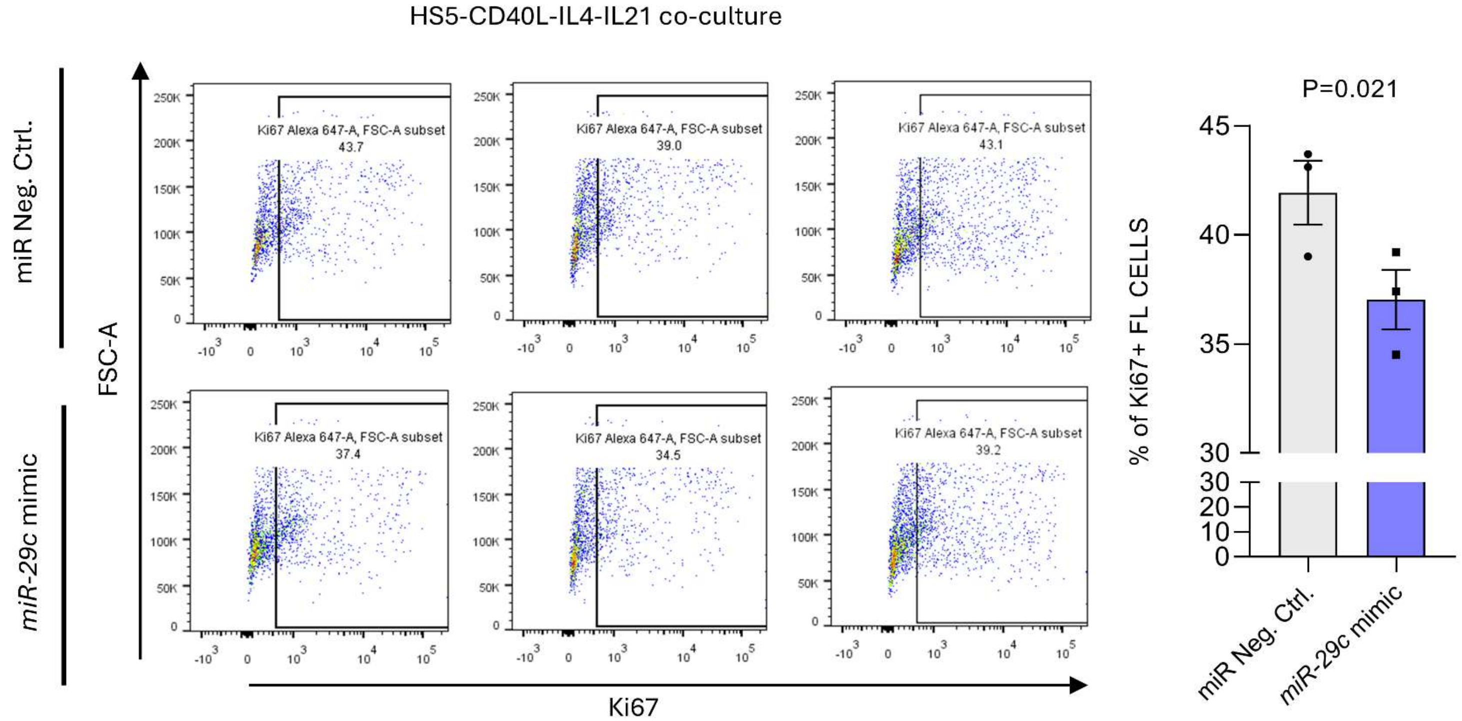

Figure S13: Left: Intracellular Ki67 staining in primary B-cells from FL lymph node transfected with synthetic *miR-29c* mimic or negative control (miR Neg. Ctrl.) and cocultured (96 h) with HS5-CD40L-IL4-IL21 cells. The TRAF4 downmodulation by *miR-29c* in this experiment has not been quantified by immunoblot due to a very limited amount of material available (primary FL cells)**. Right: Statistical analysis of percentage of Ki67+ cells (paired t-test).** The error bars indicate SEM.

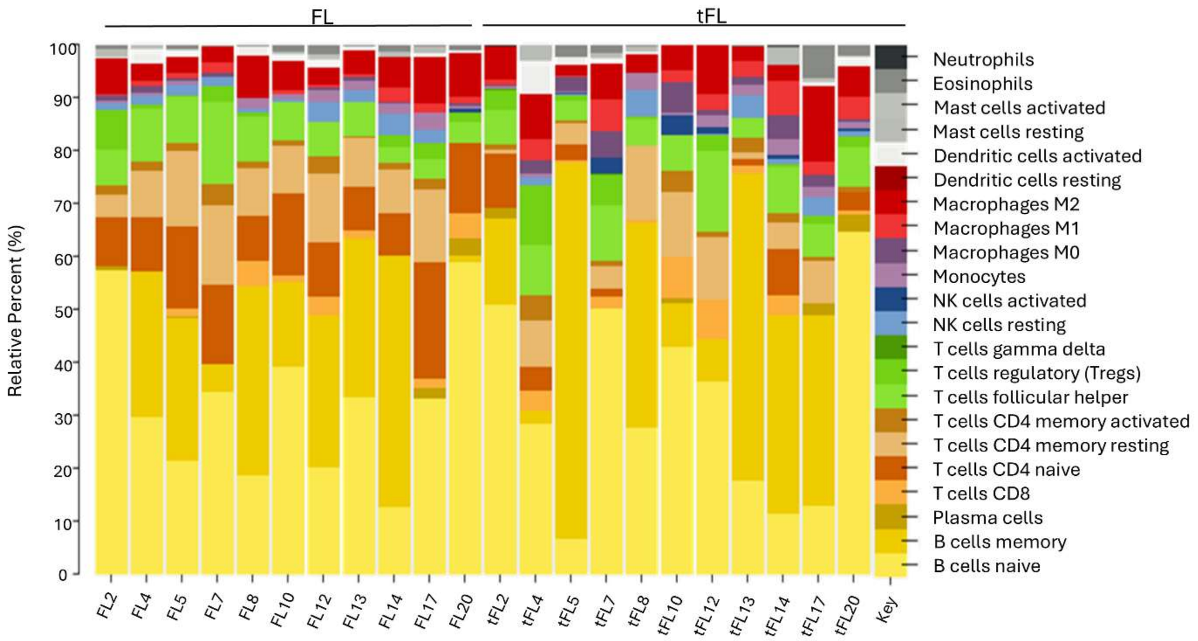

Figure S14: Overview of cell populations (CIBERSORTx) identified from bulk mRNA-seq of paired FL-tFL samples (n=11 pairs; RNA-seq in this study).

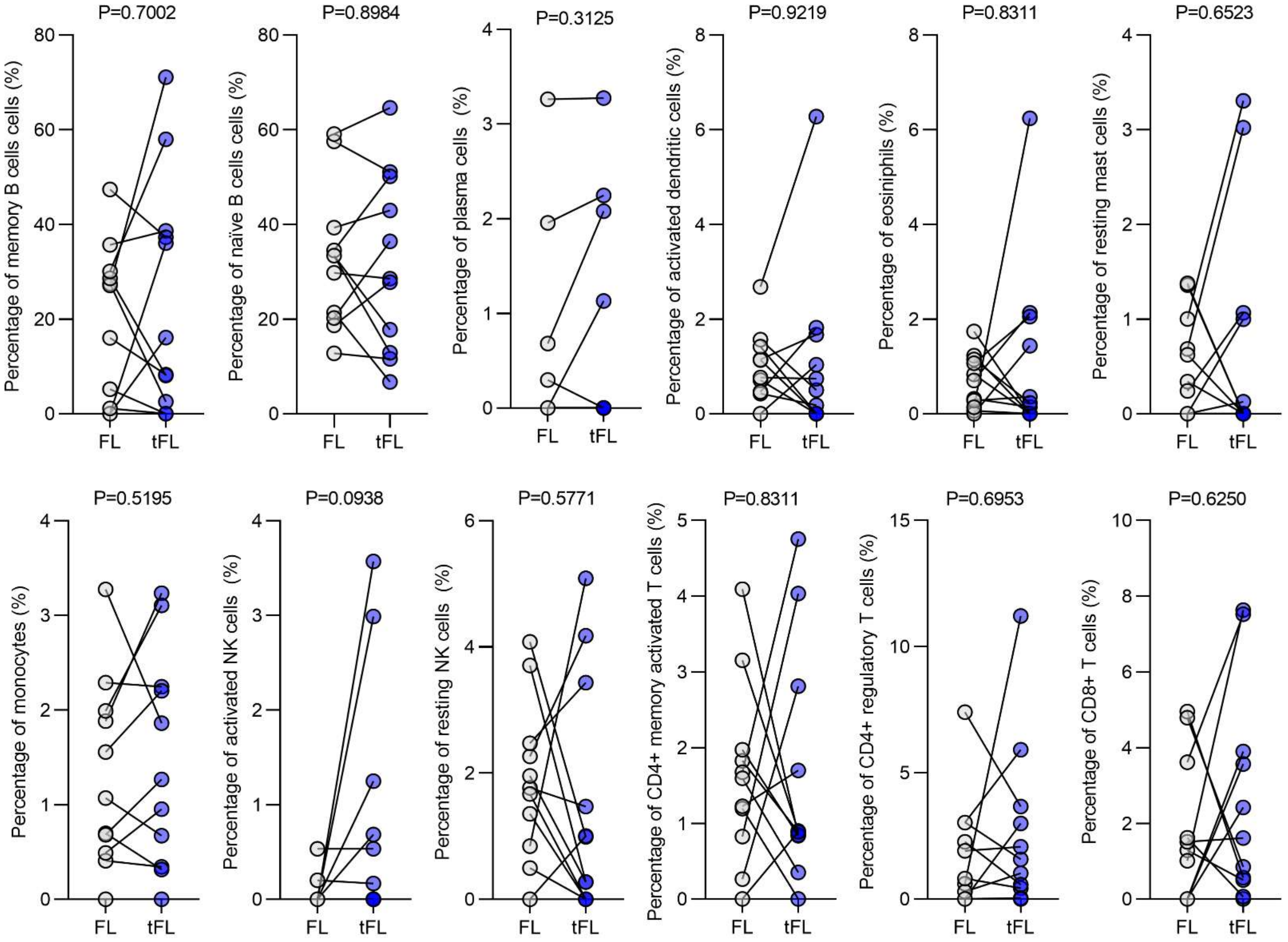

Figure S15: CIBERSORTx analysis of cell populations from bulk mRNA-seq of paired FL-tFL samples (n=11 pairs; RNA-seq in this study). This figure shows cell types not previously shown in Figure 4C. Resting dendritic cells, activated mast cells, neutrophils, and gamma delta T cells are not visualized since they were undetectable in most samples. Statistical differences were compared using the Wilcoxon matched paired test

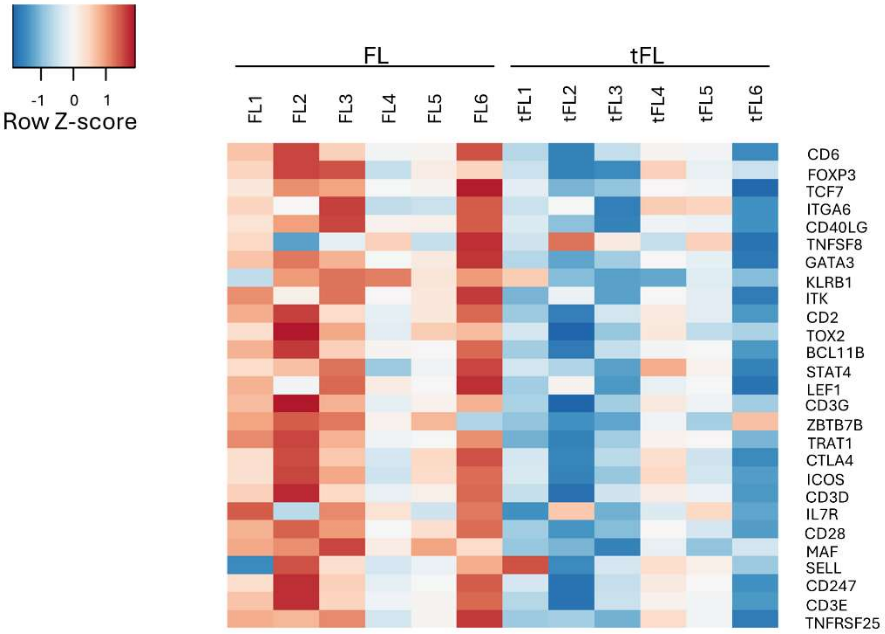

Figure S16: Expression of T-cell specific markers (signature from ref.[31,32]) in fresh-frozen FL-tFL mRNA-seq data from Parsa et al.[19] (n=6 pairs) visualized as heatmap. Row z-score from normalized counts for each mRNA was plotted.

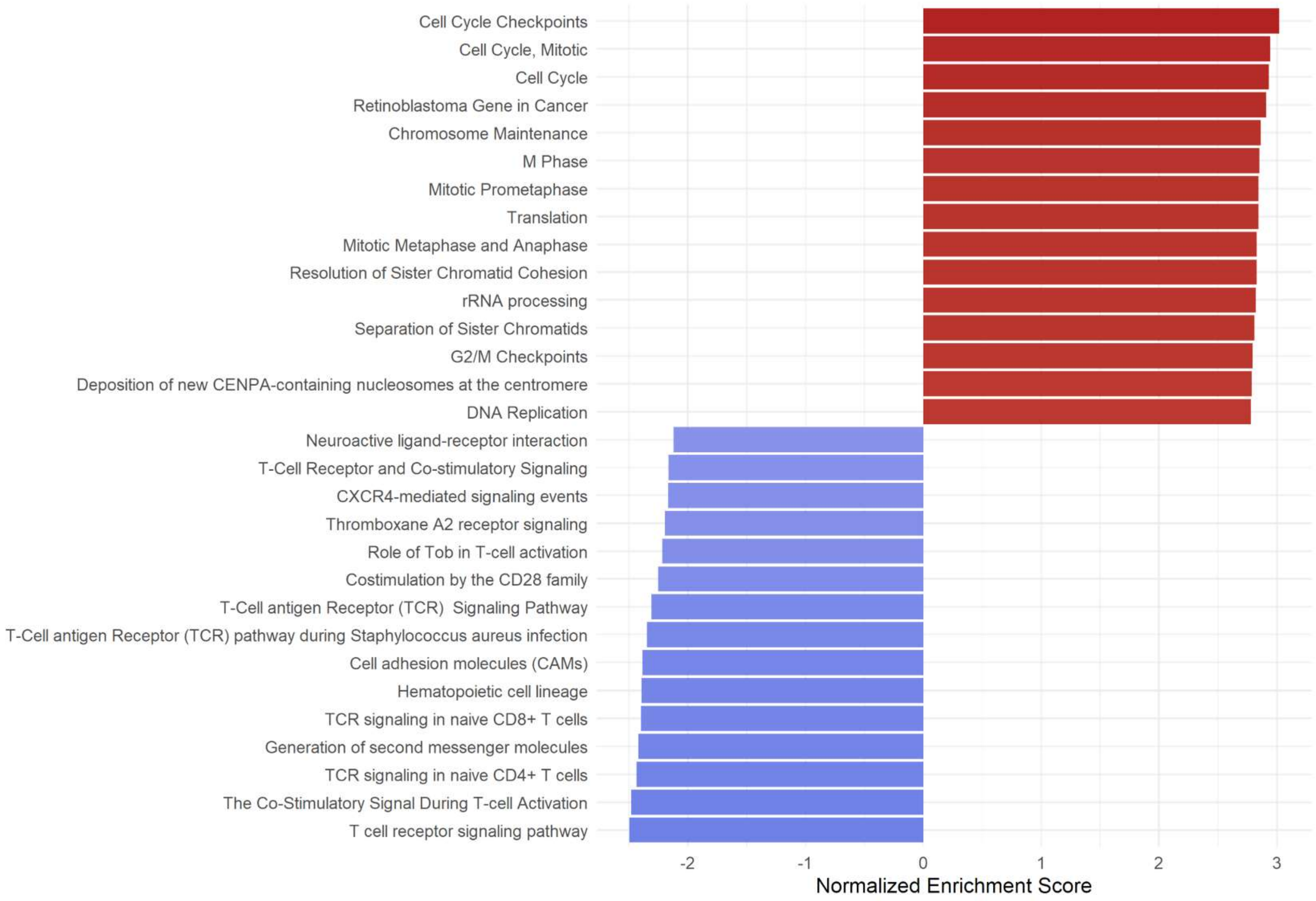

Figure S17: GSEA analysis highlighting pathways and biological processes changed in our FL-tFL mRNA-seq data (n=11 pairs; RNA-seq in this study). Red color represents an increase in the activity of the pathway, while blue represents a decrease.

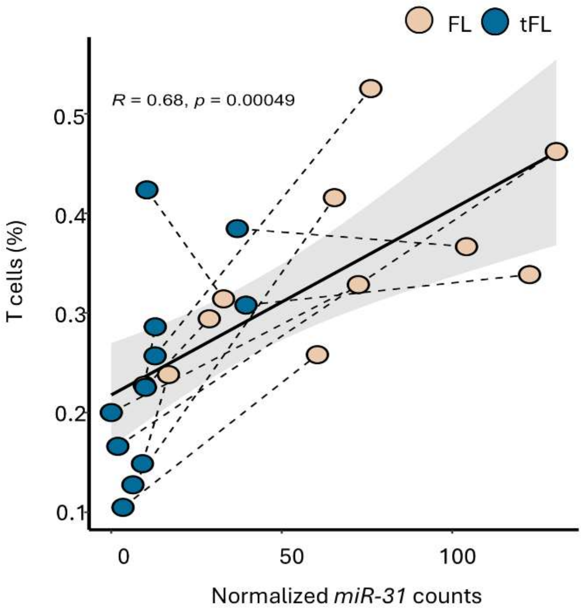

Figure S18: Pearson correlation of T cell percentage (CIBERSORTx tool) obtained from analysis of bulk mRNAs-seq of FL-tFL (n=11 pairs; RNA-seq in this study) and *miR-31* expression (normalized counts from miRNA-seq of 11 FL-tFL pairs).

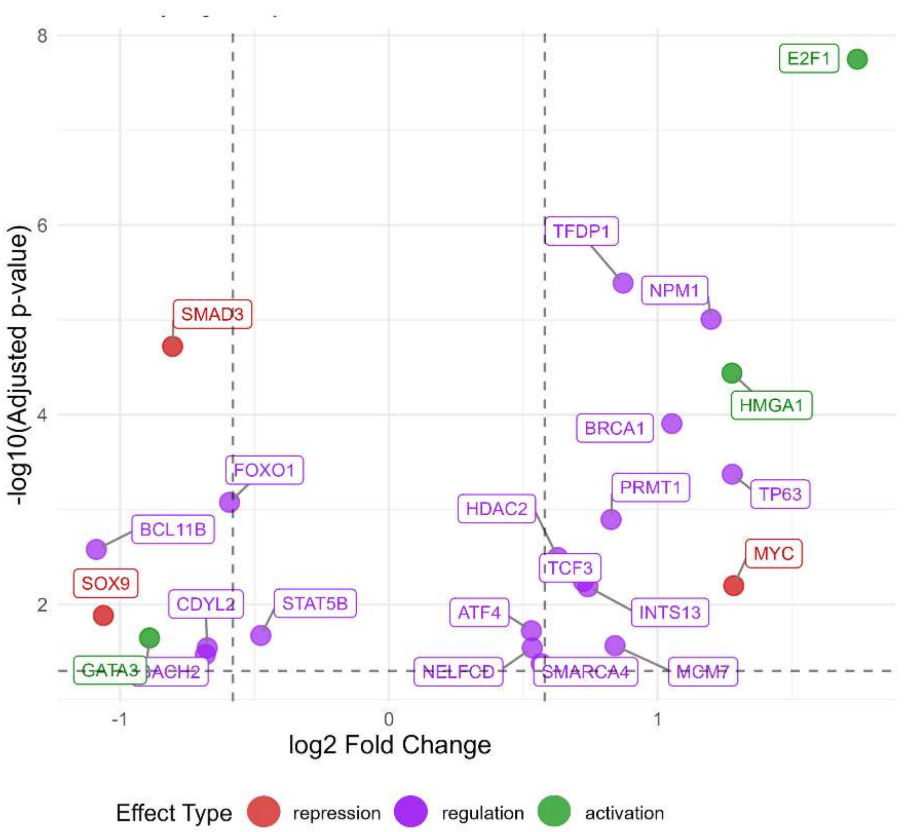

Figure S19: Volcano plot of transcription factors that regulate expression of *miR-29* family according to Transmir 3.0[36] database and are significantly expressed in our mRNA-seq FL-tFL data. Red color represents transcription factors that repress expression of their miRNA while green color represents those that activate according to literature. Purple color represents transcription factors with evidence for binding without known direction of effect.

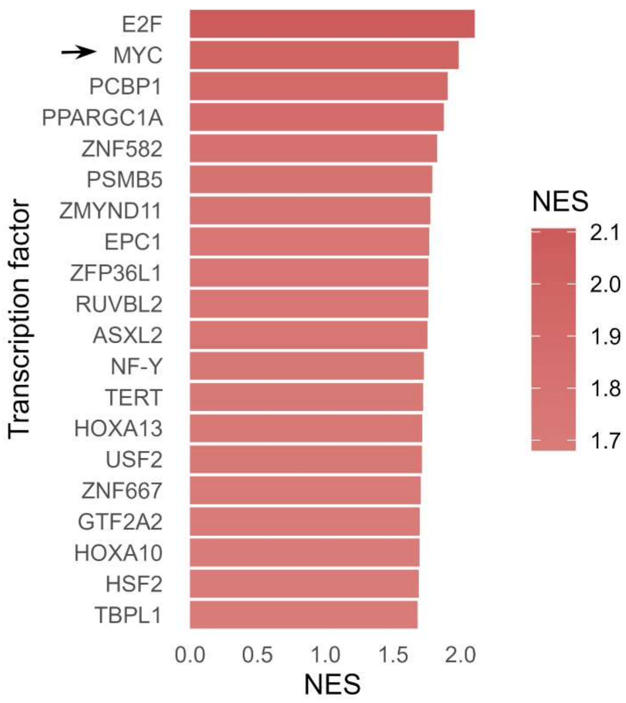

Figure S20: GSEA analysis highlighting changes in the activity of transcription factors according to mRNA-seq of paired FL-tFL samples (n=11 pairs; RNA-seq in this study).

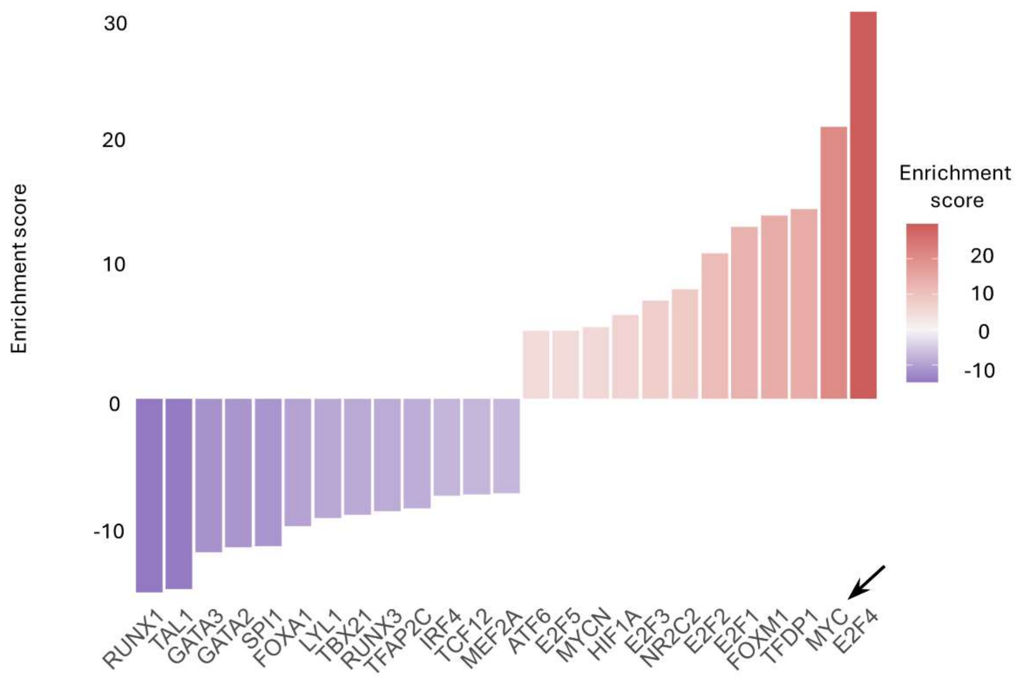

Figure S21: Analysis of transcription factor activity with decoupleR with DoRothEA tool according to mRNA-seq of paired FL-tFL samples (n=11 pairs). The red color represents an increase in transcription factor activity upon FL transformation.

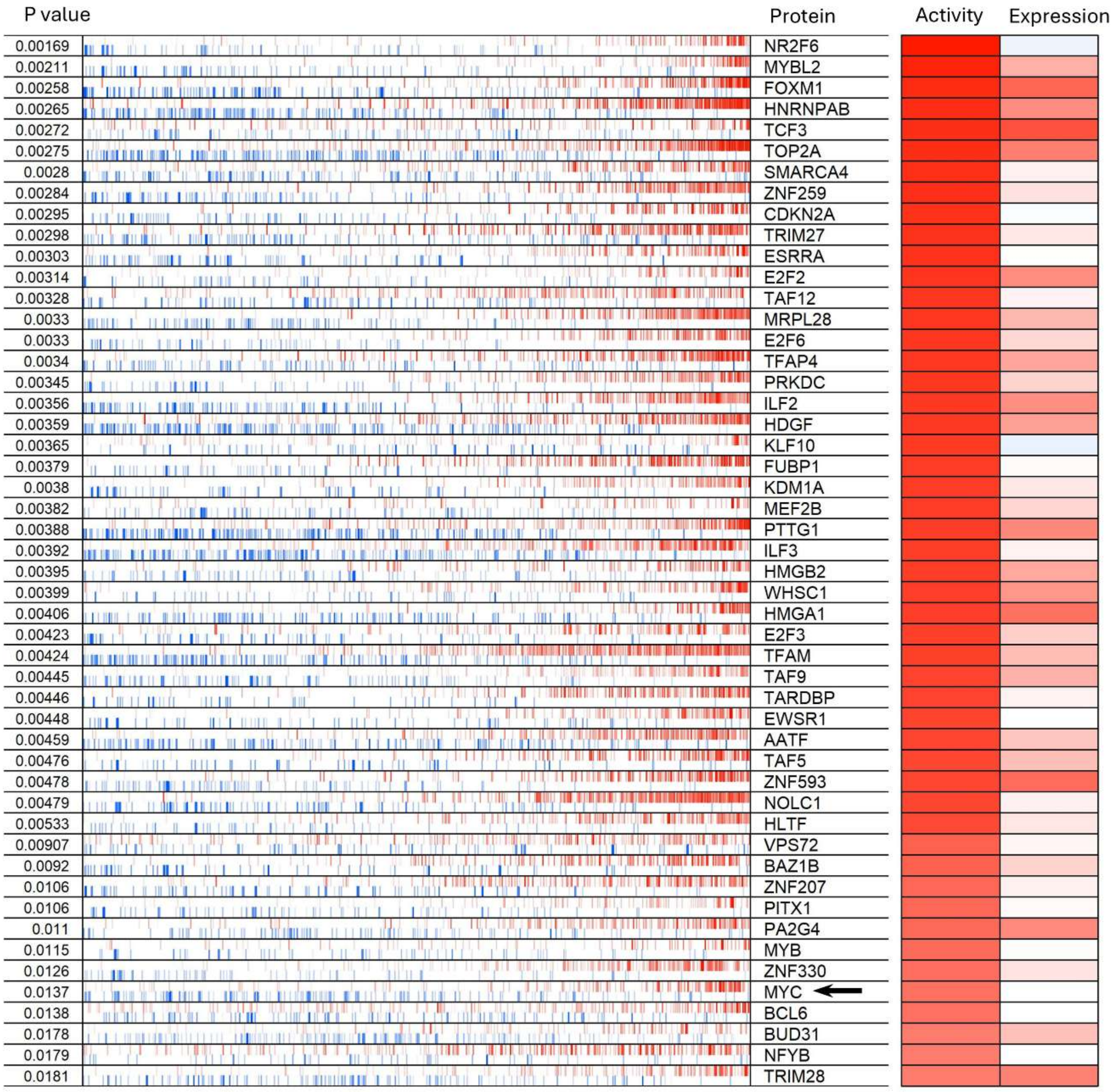

Figure S22: VIPER analysis of mRNA-seq data from fresh-frozen FL-tFL samples (data from Parsa et al.[19], n=6 pairs), depicting the top 50 transcription factors with increased activity in tFL. The red color represents an increase in activity.

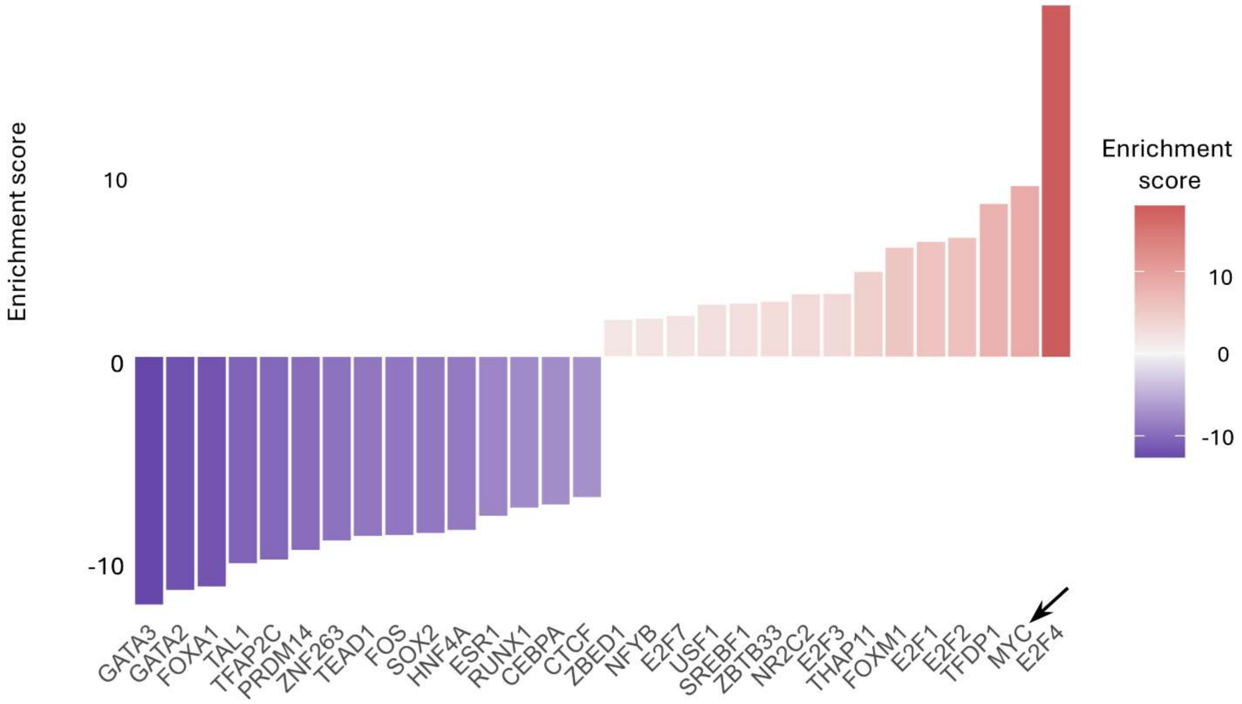

Figure S23: Analysis of transcription factor activity with decoupleR with DoRothEA tool for mRNA-seq data from fresh-frozen FL-tFL samples (data from Parsa et al.[19], n=6 pairs). The red color represents an increase in upstream regulator/ pathway activity, and the blue represents a decrease.

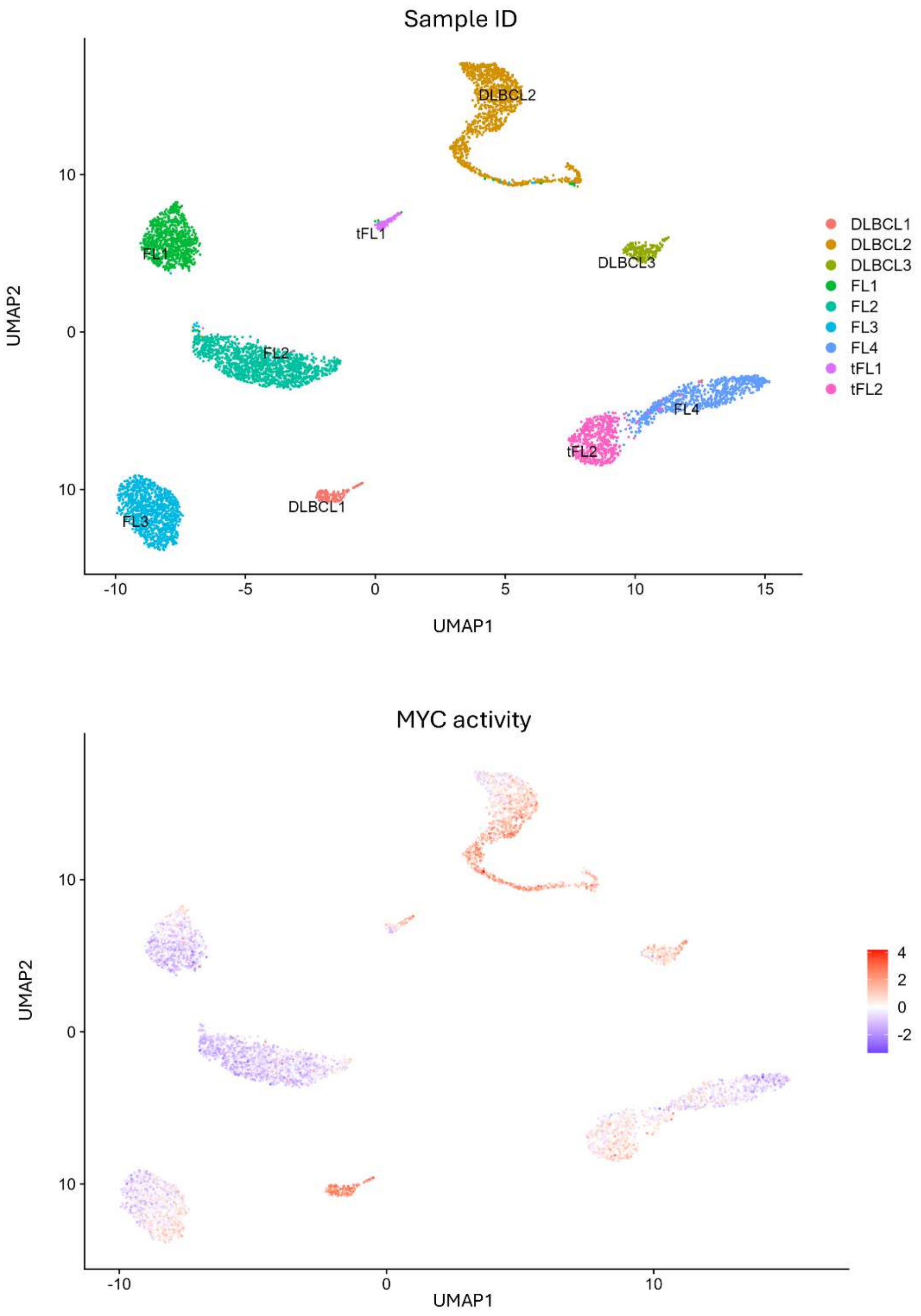

Figure S24: decoupleR with DoRothEA analysis of MYC activity in scRNA-seq data from malignant B-cells from FL (n=4), tFL (n=2), and DLBCL (n=3) samples (data from Roider et al.[21]). Top section represents annotation for individual samples, and bottom part represents MYC activity (red color represents increase in activity and blue color represents decrease in activity).

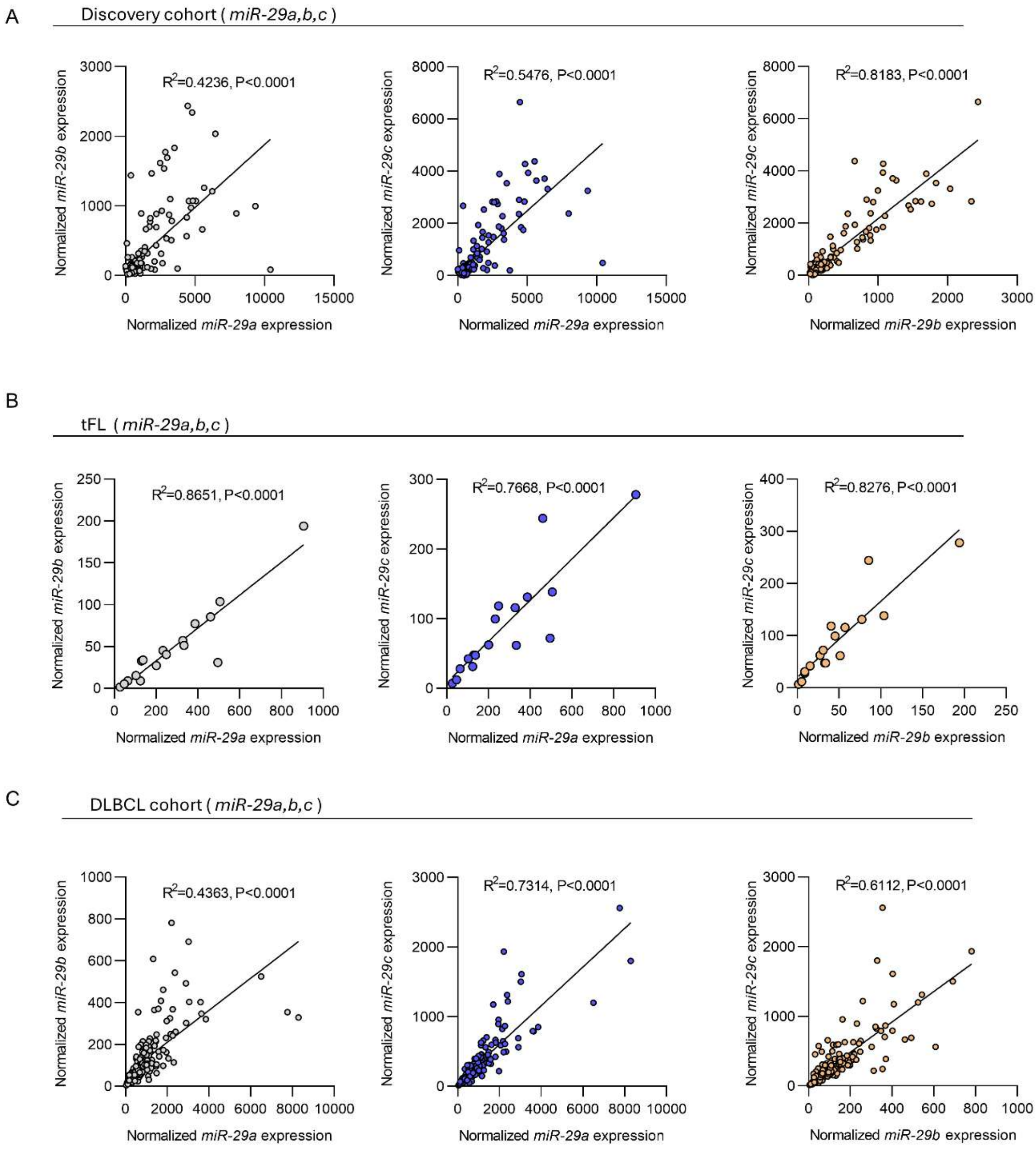

Figure S25: Correlation of *miR-29s* expression in (A) discovery cohort (n=185), (B) tFL sample cohort (n=17) and (C) DLBCL cohorts (n=171).

Discovery cohort (*miR-29a,b,c*)-OS

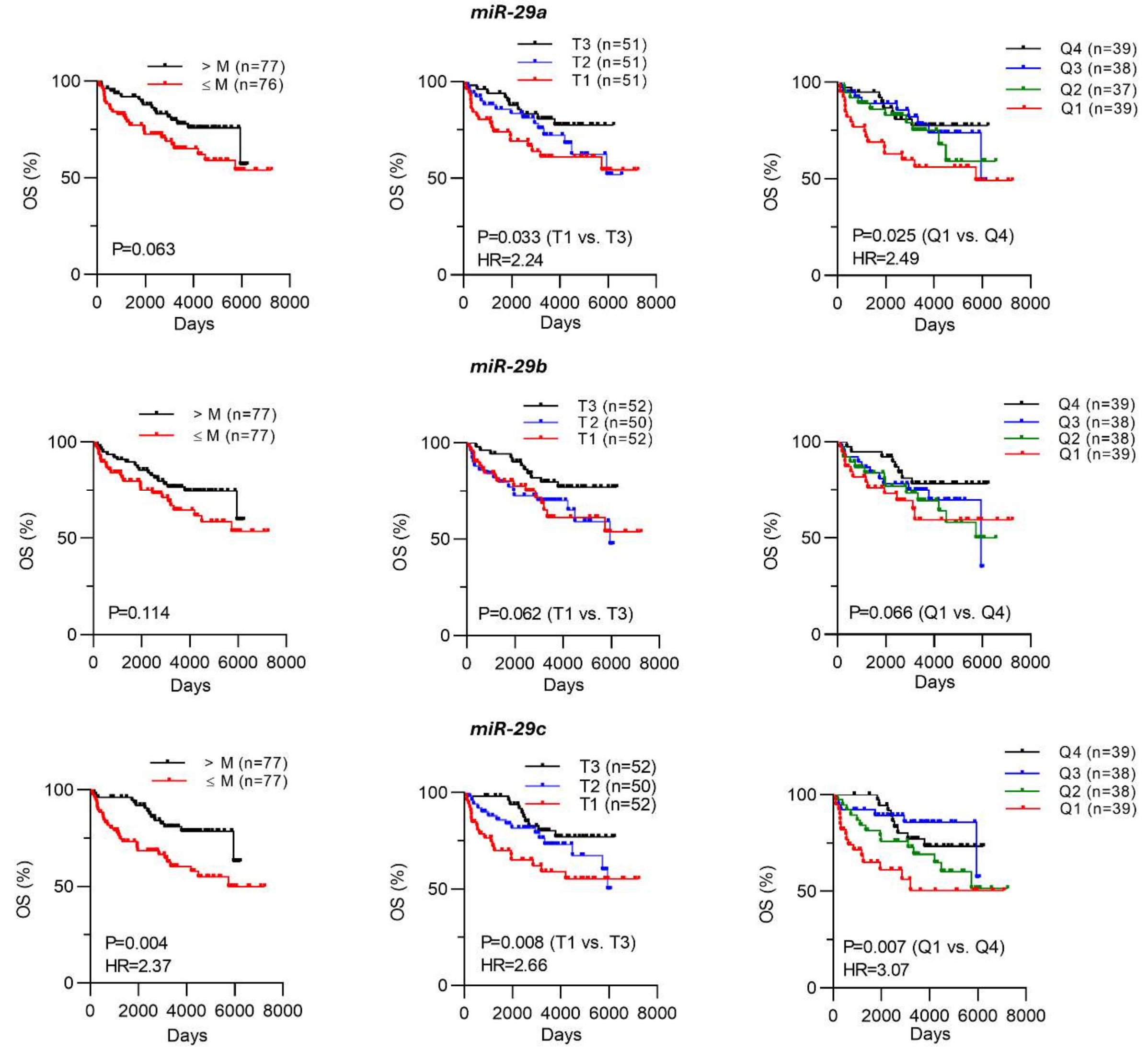

Figure S26: Association of *miR-29* family expression with OS in FL patients (n=154) consisting only of samples obtained at diagnosis. The expression level of *miR-29a* was not available for 1 sample because of technical issues with miRNA quantification. Patients were separated based on the median, tercile, and quartile of *miR-29a/b/c* expression. Expression of *miR-29a/b/c* was normalized to expression of *RNU38B.*

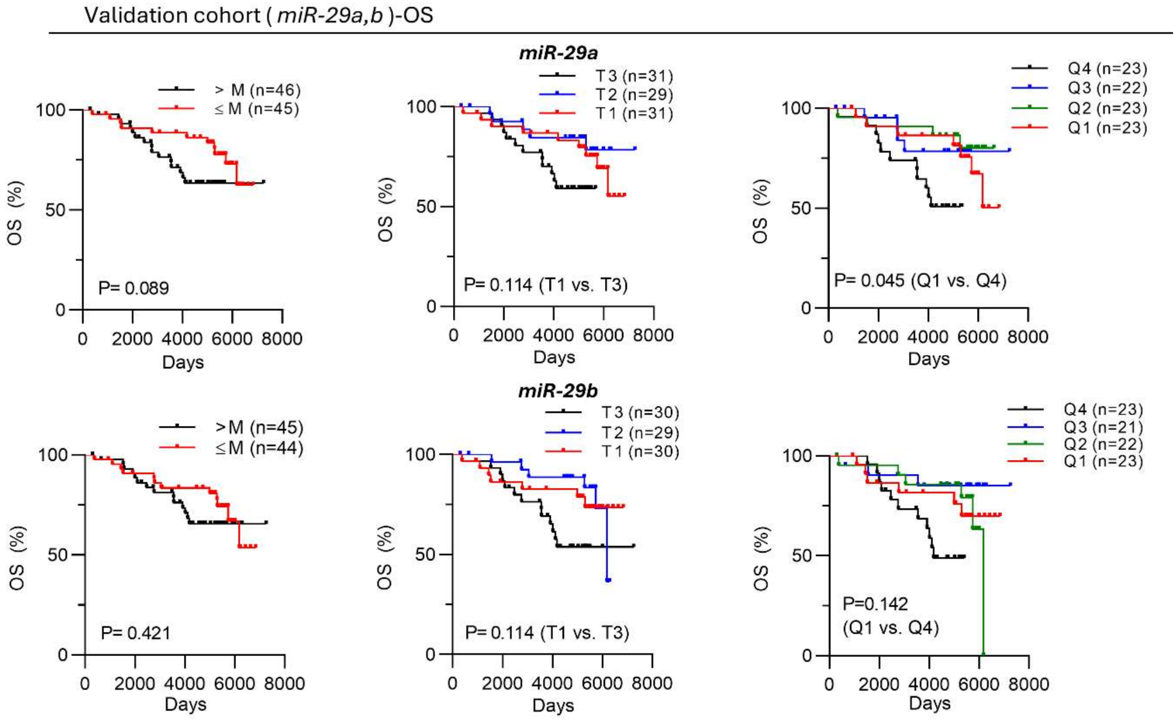

Figure S27: Association of *miR-29a* and *miR-29b* expression with OS in validation SWOG S0016 cohort (NCT00006721[37]) **of FL patients (n=91 for *miR-29a* and n=89 for *miR-29b*).** The expression level of *miR-29a* and *miR-29b* was not available for 1 and 3 samples, respectively (all cohort n=92) because of technical issues with miRNA quantification. Patients were separated based on the median (M), tercile (T), and quartile (Q) of *miR-29a/b* expression. Expression of *miR-29a/b* was normalized to geometric mean of *RNU38B, RNU6B,* and *miR-16* expression.

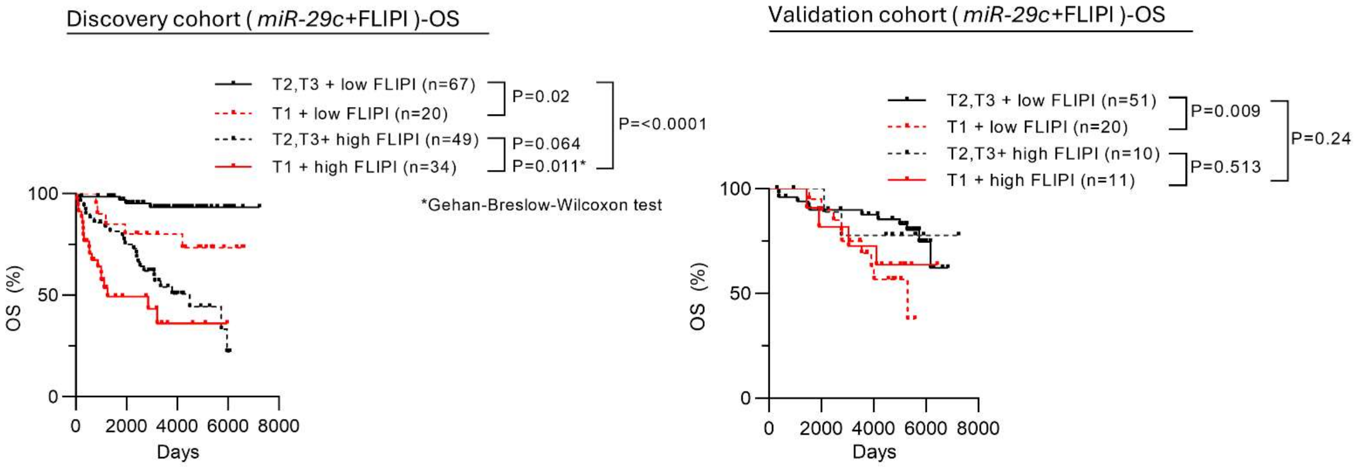

Figure S28: Association of *miR-29c* expression combined with FLIPI with PFS in the discovery and validation cohort. Patients were dichotomized based on the tercile (T1 vs. T2+3) of *miR-31* expression and high vs. low FLIPI. Expression of *miR-29c* was normalized to expression of *RNU38B* in Discovery cohort and to geometric mean of *RNU38B, RNU6B,* and *miR-16* expression in Validation cohort.

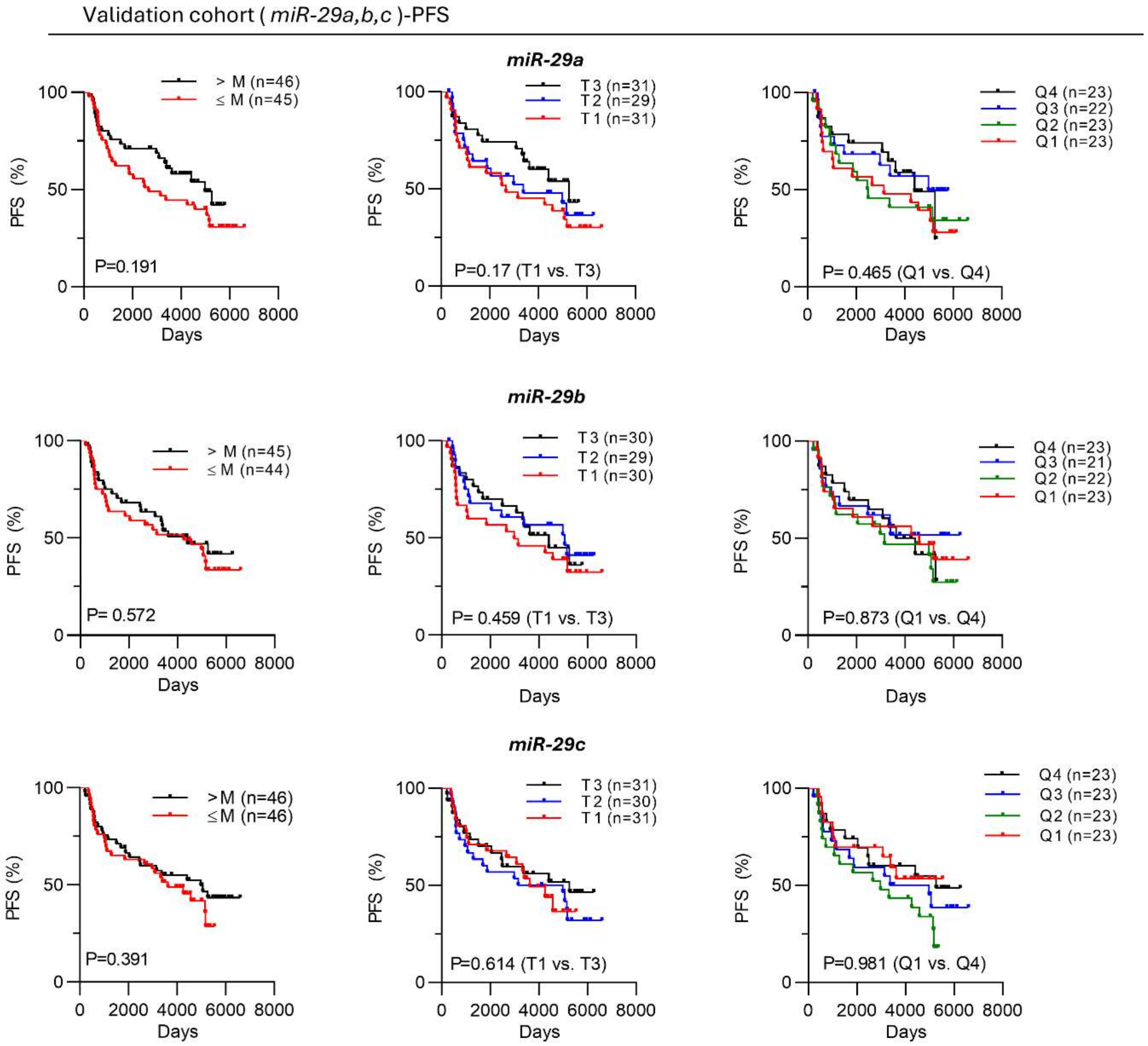

Figure S29: Association of *miR-29a, miR-29b,* and *miR-29c* expression with PFS in a validation SWOG S0016 (NCT00006721[37]**) cohort of FL patients (n=91 for *miR-29a* and n=89 for *miR-29b*).** The expression level of *miR-29a* and *miR-29b* was not available for 1 and 3 samples, respectively (all cohort n=92) because of technical issues with its quantification. Patients were separated based on the median (M), tercile (T), and quartile (Q) of *miR-29a/b/c* expression. Expression of *miR-29a/b/c* was normalized to geometric mean of *RNU38B, RNU6B,* and *miR-16* expression

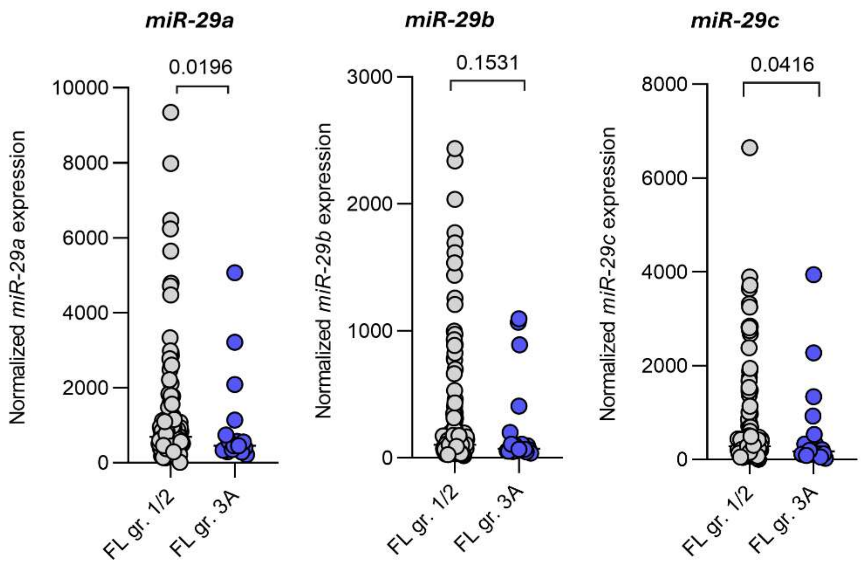

Figure S30: Association of *miR-29a/b/c* with grade (grade 1 and 2 vs. grade 3) in discovery cohort of FL patients (n=154) consisting only of samples obtained at diagnosis. The *miR-29a* expression level was not available for 1 sample because of technical issues with its quantification. We did not have information about grade for 5 samples. Statistical differences were compared by Mann-Whitney test.

DLBCL cohort (*miR-29a,b,c*)-OS

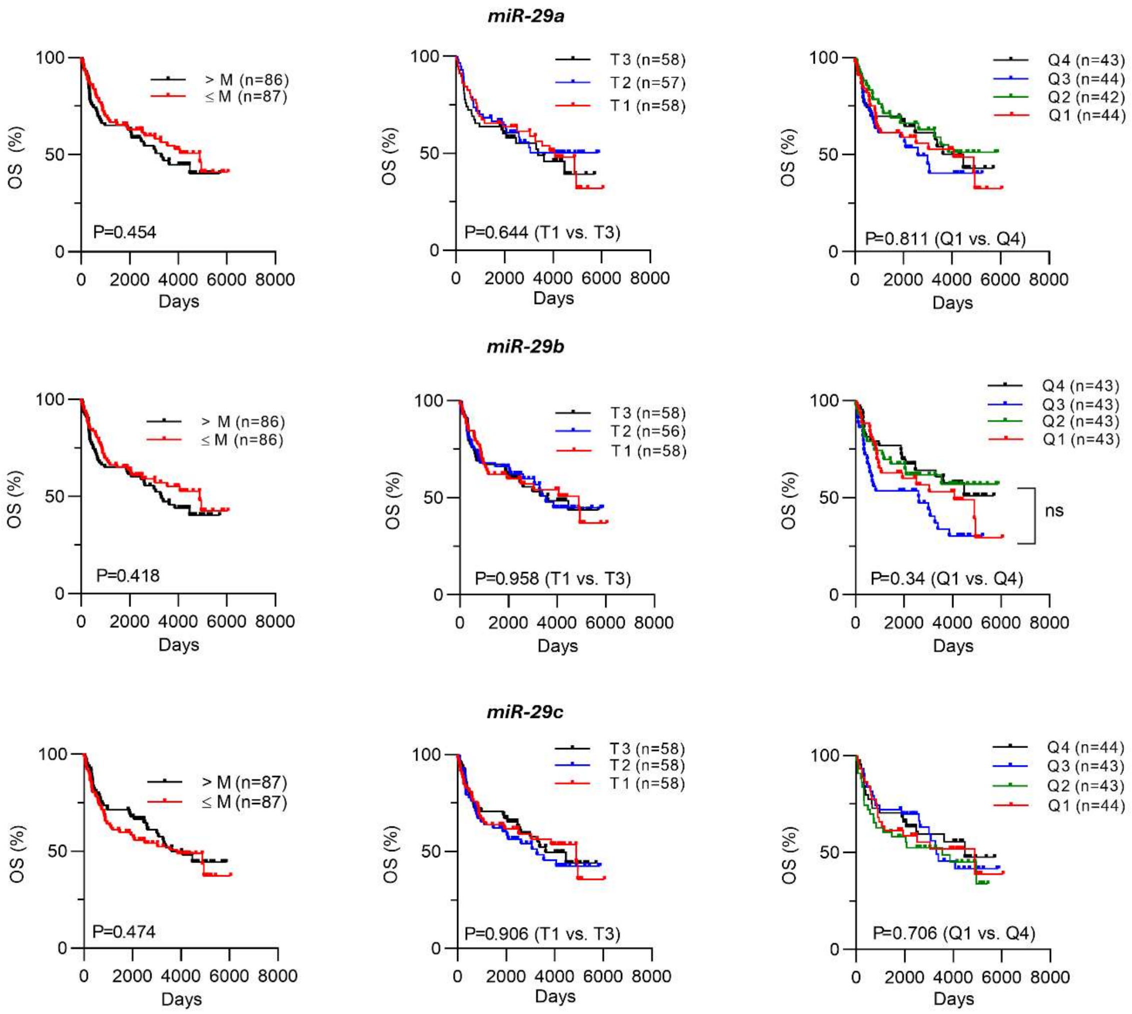

Figure S31: Association of *miR-29a, miR-29b,* and *miR-29c* expression with OS in a de novo DLBCL patients (n=174) cohort. Patients were separated based on the median (M), tercile (T), and quartiles (Q) of *miR-29a/b/c* expression. Expression of *miR-29a/b/c* was normalized to expression of *RNU38B.*

DLBCL cohort (*miR-29a,b,c*)-PFS

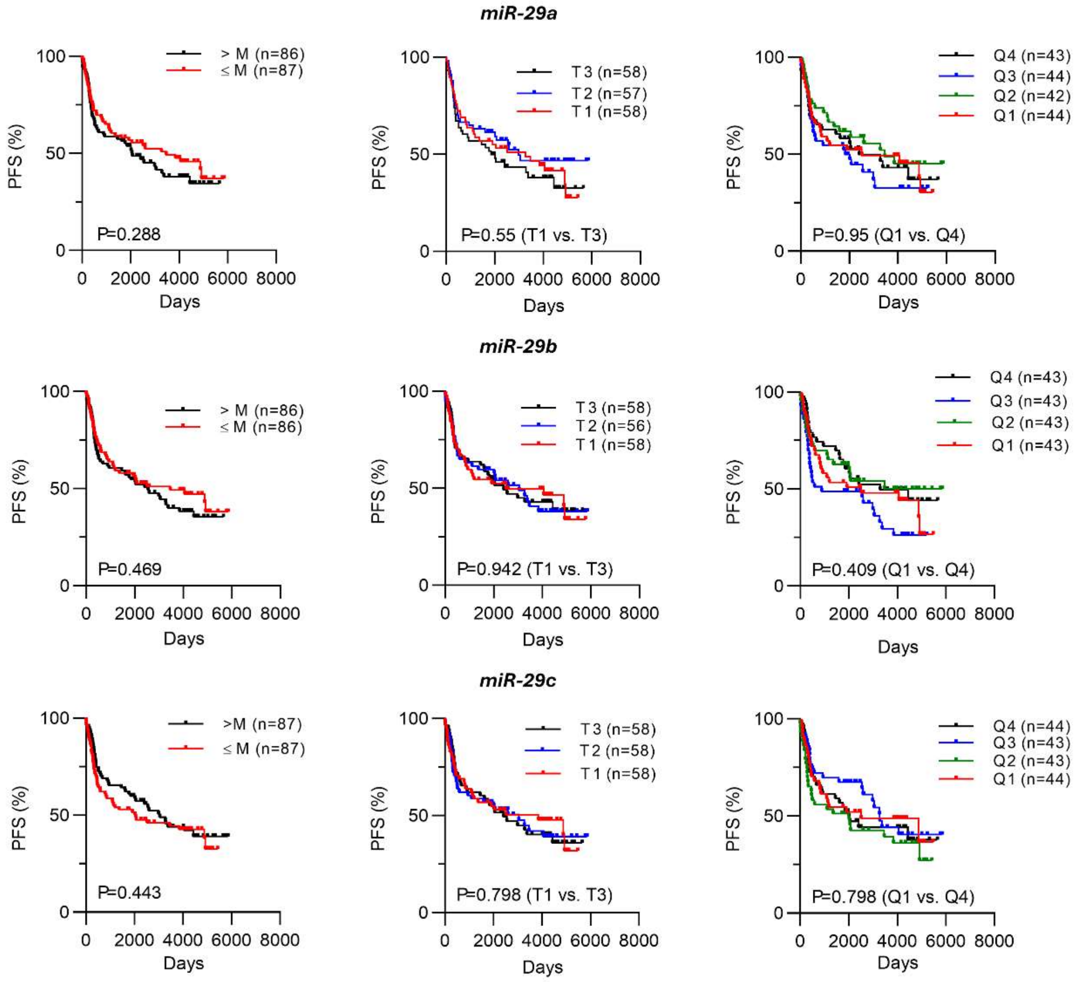

Figure S32: Association of *miR-29a, miR-29b,* and *miR-29c* expression with PFS in a cohort of *de novo* DLBCL patients (n=174). Patients were separated based on the median (M), tercile (T), and quartile (Q) of *miR-29a/b/c* expression. Expression of *miR-29a/b/c* was normalized to expression of *RNU38B.*

A

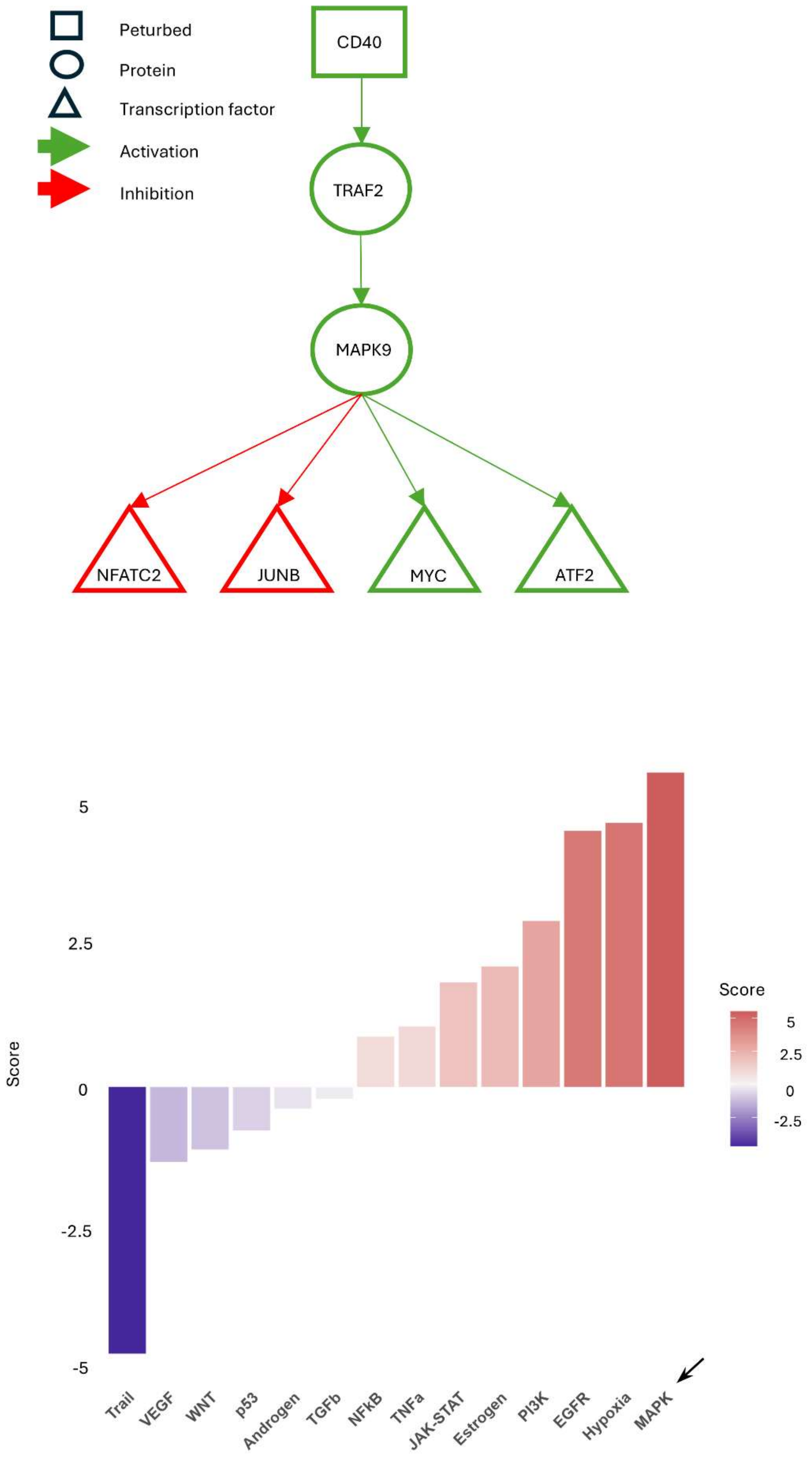

Figure S33: (A) Causal reasoning-based upstream network analysis (CARNIVAL tool) starting from CD40 using mRNA-seq data from paired FL-tFL samples (n=11 pairs; RNA-seq in this study). (B) Analysis of pathway activity from mRNA-seq of paired FL-tFL samples (n=11 pairs) using decoupleR with PROGENy. Red color represents an increase in the activity of the pathway, while blue represents a decrease.

3) Supplemental Tables

Supplemental Table 1. Characteristics of paired FL and tFL samples used for miRNA-seq and mRNA-seq

Patient no.	Lymphoma type	ID	Gender	Age	Histological evaluation at the time of biopsy	Time of biopsy	Time from diagnosis to transformation [months]	Immunoglobulin rearrangement*	MYC aberration	Usage
**2**	FL	2FL	F	60	FL G2	at relapse	65	IGHV1-18*01/IGHD3-10*02/IGHJ4*02		miRNAseq & mRNAseq
**2**	tFL	2tFL	F	61	DLBCL	at transformation		IGHV1-18*01/IGHD3-10*02/IGHJ4*02	N.D.	miRNAseq & mRNAseq
**4**	FL	4FL	F	55	FL	at diagnosis	23	IGHV3-7*01/IGHD3-10*01/IGHJ4*02		miRNAseq & mRNAseq
**4**	tFL	4tFL	F	57	DLBCL (60%) /FL (40%)	at transformation		IGHV3-7*01/IGHD3-10*01/IGHJ4*02	no translocation#	miRNAseq & mRNAseq
**5**	FL	5FL	M	57	FL G3A	at diagnosis	73	N.D.		miRNAseq & mRNAseq
**5**	tFL	5tFL	M	63	DLBCL (35%) / FL G3B (65%)	at transformation		N.D.	no translocation, no amplification	miRNAseq & mRNAseq
**7**	FL	7FL	F	70	FL G1-2	at diagnosis	19	IGLV3-21*02/IGLJ1*01		miRNAseq & mRNAseq
**7**	tFL	7tFL	F	72	DLBCL	at transformation		IGLV3-21*02/IGLJ1*01	translocation	miRNAseq & mRNAseq
**8**	FL	8FL	M	61	FL G3A	at diagnosis	8	IGHV2-70*XX/IGHD3-16*01/IGHJ4*02		miRNAseq & mRNAseq
**8**	tFL	8tFL	M	62	DLBCL/BL	at transformation		N.D.	translocation	miRNAseq & mRNAseq
**10**	FL	10FL	F	56	FL G1	at diagnosis	96	IGHV3-21*XX/IGHD6-6*01/IGHJ4*02		mRNAseq
**10**	tFL	10tFL	F	64	DLBCL	at transformation		IGHV3-21*XX/IGHD6-6*01/IGHJ4*02	no translocation, no amplification	mRNAseq
**12**	FL	12FL	M	72	FL G1	at diagnosis	106	IGKV1-39*01/J4*01		miRNAseq & mRNAseq
**12**	tFL	12tFL	M	80	DLBCL / FL G3A (<20%)	at transformation		IGKV1-39*01/J4*01	translocation	miRNAseq & mRNAseq
**13**	FL	13FL	F	52	FL G1-2 (focal G3)	at diagnosis	52	IGHV4-59*04/IGHD2-21*02/IGHJ4*02		miRNAseq & mRNAseq
**13**	tFL	13tFL	F	57	DLBCL	at transformation		IGHV4-59*04/IGHD2-21*02/IGHJ4*02	N.D.	miRNAseq & mRNAseq
**14**	FL	14FL	F	64	FL G2	at diagnosis	23	N.D.		miRNAseq & mRNAseq
**14**	tFL	14tFL	F	66	DLBCL	at transformation		N.D.	N.D.	miRNAseq & mRNAseq
**15**	FL	15FL	M	69	FL G2	at diagnosis	7	N.D.		miRNAseq
**15**	tFL	15tFL	M	70	DLBCL	at transformation		N.D.	no translocation	miRNAseq
**17**	FL	17FL	M	70	FL G2	at relapse	116	N.D.		mRNAseq
**17**	tFL	17tFL	M	75	DLBCL	at transformation		N.D.	no translocation	mRNAseq
**20**	FL	20FL	M	63	FL G2	at diagnosis	8	N.D.		miRNAseq & mRNAseq
**20**	tFL	20tFL	M	64	DLBCL	at transformation		N.D.	N.D.	miRNAseq & mRNAseq

* We provide immunoglobulin gene rearrangement data for the heavy or light chains based on which product was reliably detected by PCR.

# No evidence of MYC (8q24) abnormality based on karyotyping (FISH not done).

DLBCL, diffuse large B-cell lymphoma; FL, follicular lymphoma; tFL, transformed follicular lymphoma; G1, grade 1; G2, grade 2; G3, grade 3; N/A, not analyzed; N.D., not determined due to insufficient quality of input material.

Supplemental Table 2. Clinical characteristics of Discovery FL cohort (n=185)		
	No.	[%]
**Gender**		
Female	96	51.9
Male	89	48.1
**Age**		
≤60	102	55.1
> 60	83	44.9
**median (min–max):**	59 (33–88)	
**Grade**		
1	81	43.8
2	53	28.6
1–2	19	10.3
2–3A	6	3.2
3A	16	8.6
Not determined	10	5.4
**Stage**		
I	13	7.0
II	30	16.2
III	55	29.7
IV	79	42.7
Not determined	8	4.3
**B symptoms**		
Absent	127	68.6
Present	52	28.1
Not determined	6	3.2
**Nodal sites**		
≤4	95	51.4
> 4	80	43.2
Not determined	10	5.4
**Extranodal sites**		
0	89	48.1
≥ 1	82	44.3
Not determined	14	7.6
**ECOG**		
0	108	58.4
1	54	29.2
2	14	7.6
Not determined	9	4.9
**FLIPI**		
Low (0-1)	41	22.2
Intermediate (2)	46	24.9
High (3-5)	83	44.9
Not determined	15	8.1
**OS Follow-up [days]**		
median (min–max):	3276 (45–7415)	
**PFS Follow-up [days]**		
median (min–max):	1561 (24–7198)	
**First line therapy**		
R-CHOP	110	59.5
R-COP(P) or R-FND	16	8.6
Radiotherapy only	8	4.3
Rituximab only	6	3.2
CHOP or FND	28	15.1
Other	17	9.2

**Supplementary Table 3. Clinical characteristics of Validation FL (SWOG 0016) cohort (n=92)**		
	No.	[%]
**Age**		
≤ 60	65	70.7
> 60	27	29.3
**median (min–max):**	54 (33–88)	
**Grade**		
1–2	87	94.6
3	4	4.3
Not determined	1	1.1
**Stage**		
II	3	3.3
III	40	43.5
IV	49	53.3
**B symptoms**		
Absent	64	69.6
Present	28	30.4
**FLIPI**		
Low (0-1)	28	30.4
Intermediate (2)	43	46.7
High (3-4)	21	22.8
**OS Follow-up [days]**		
median (min–max):	5063 (302–7248)	
**PFS Follow-up [days]**		
median (min–max):	3416 (243–7248)	
**First line therapy**		
R-CHOP	92	100.0

Supplemental Table 4. Clinical characteristics of DLBCL cohort (n=174)		
	No.	[%]
**Gender**		
Female	84	48.3
Male	90	51.7
**Age**		
≤60	68	39.1
> 60	106	60.9
**median (min–max):**	67 (17–88)	
**Stage**		
I	23	13.2
II	57	32.8
III	40	23.0
IV	53	30.5
Not determined	1	0.6
**B symptoms**		
Absent	84	48.3
Present	87	50.0
Not determined	3	1.7
**IPI**		
Low (0-1)	41	23.6
Intermediate (2-3)	91	52.3
High (4-5)	38	21.8
Not determined	4	2.3
**AA IPI**		
Low (0)	20	11.5
Intermediate (1-2)	113	64.9
High (3)	39	22.4
Not determined	2	1.1
**OS Follow-up [days]**		
**median (min–max):**	2274 (11–6048)	
**PFS Follow-up [days]**		
**median (min–max):**	1975 (7–5863)	
**First line therapy**		
R-CHOP/R-CHOP-like	125	71.8
R-CHOP/R-CHOP-like + Radiotherapy	22	12.6
R-CHOP/R-CHOP-like +systemic CNS prophylaxis	22	12.6
Other	5	2.9

**Supplementary Table 5a**: Univariate Cox regression analyses of **OS** for Discovery cohort.				
Univariate Cox Proportional Hazard Regression:				
**miR-29a**	**P value**	**Hazard Ratio (HR)**	**95% HR Lower CI**	**95% HR Upper CI**
miR-29a levels ≤median	0.009	2.09	1.20	3.66
miR-29a levels < 1st tercile	0.008	2.05	1.21	3.49
miR-29a levels < 1st quartile	0.009	2.07	1.20	3.58
**miR-29b**				
miR-29b levels ≤median	0.016	1.95	1.13	3.35
miR-29b levels < 1st tercile	0.074	1.63	0.95	2.78
miR-29b levels < 1st quartile	0.065	1.70	0.97	2.98
**miR-29c**				
miR-29c levels ≤median	0.001	2.50	1.42	4.39
miR-29c levels < 1st tercile	0.014	1.94	1.14	3.30
miR-29c levels < 1st quartile	0.017	1.97	1.13	3.44
FLIPI > 2	<0.001	6.28	3.35	11.77
Ann Arbor Stage III-IV	0.024	2.28	1.12	4.67
Age > 60 years	0.000	3.88	2.17	6.91
B symptoms present	0.038	1.77	1.03	3.05
LDH elevated	<0.001	3.69	1.95	7.01
Hemoglobin <120 g/L	0.010	2.11	1.19	3.73

**Supplementary Table 5b**: Univariate Cox regression analyses of **PFS** for Discovery cohort.				
Univariate Cox Proportional Hazard Regression:				
**miR-29a**	**P value**	**Hazard Ratio (HR)**	**95% HR Lower CI**	**95% HR Upper CI**
miR-29a levels ≤median	0.054	1.53	0.99	2.36
miR-29a levels < 1st tercile	0.116	1.44	0.91	2.25
miR-29a levels < 1st quartile	0.012	1.85	1.15	2.97
**miR-29b**				
miR-29b levels ≤median	0.157	1.36	0.89	2.09
miR-29b levels < 1st tercile	0.323	1.26	0.80	1.97
miR-29b levels < 1st quartile	0.360	1.26	0.77	2.04
**miR-29c**				
miR-29c levels ≤median	0.018	1.68	1.09	2.59
miR-29c levels < 1st tercile	0.005	1.88	1.21	2.91
miR-29c levels < 1st quartile	0.071	1.57	0.96	2.56
FLIPI > 2	<0.001	3.45	2.19	5.44
Ann Arbor Stage III-IV	0.031	1.92	1.06	3.47
Age > 60 years	0.001	2.13	1.38	3.29
B symptoms present	0.026	1.67	1.06	2.61
LDH elevated	0.008	1.84	1.17	2.88
Hemoglobin <120 g/L	0.059	1.58	0.98	2.54

**Supplementary Table 5c**: Multivariate Cox regression analyses of **OS** for Discovery cohort. Multivariate analysis included FLIPI, Ann Arbor stage, Age, B symptoms, LDH, Hemoglobin, and miRNA levels as variables (miRNA dichotomization selected from univariate analyses based on lowest P-value).				
Multivariate Cox Proportional Hazard Regression:				
**miR-29a**	**P value**	**Hazard Ratio (HR)**	**95% HR Lower CI**	**95% HR Upper CI**
FLIPI > 2	<0.001	4.08	1.97	8.45
Age > 60 years	0.001	2.71	1.48	4.94
LDH elevated	0.034	2.24	1.06	4.70
miR-29a levels	0.004	2.31	1.30	4.11
**miR-29b**				
LDH elevated	0.023	2.38	1.13	5.04
Age > 60 years	<0.001	2.96	1.62	5.40
FLIPI > 2	<0.001	4.13	1.97	8.67
miR-29b levels	0.001	2.60	1.47	4.61
**miR-29c**				
FLIPI > 2	<0.001	3.90	1.86	8.17
Age > 60 years	<0.001	3.01	1.65	5.51
LDH elevated	0.017	2.53	1.18	5.42
miR-29c levels	<0.001	2.75	1.56	4.85

**Supplementary Table 5d**: Multivariate Cox regression analyses of **PFS** for Discovery cohort. Multivariate analysis included FLIPI, Ann Arbor stage, Age, B symptoms, LDH, Hemoglobin. and miRNA levels as variables (miRNA dichotomization selected from univariate analyses based on lowest P-value).				
Multivariate Cox Proportional Hazard Regression:				
**miR-29a**	**P value**	**Hazard Ratio (HR)**	**95% HR Lower CI**	**95% HR Upper CI**
FLIPI > 2	<0.001	3,02	1,88	4,85
miR-29a levels	0.018	1,84	1,11	3,05
**miR-29b**				
FLIPI > 2	<0.001	3,02	1,88	4,83
Age > 60 years	0.035	1,62	1,04	2,54
miR-29b levels	0.057	1,53	0,99	2,37
**miR-29c**				
FLIPI > 2	<0.001	2,95	1,85	4,71
Age > 60 years	0.038	1,60	1,03	2,49
miR-29c levels	0.006	1,88	1,20	2,96
The left truncated Cox proportional hazards models (delayed entry models) were used for OS as the time of biopsy from diagnosis varied among patients. The left truncated Cox proportional hazards models (delayed entry models) for PFS were calculated from the initiation of therapy.<br>For univariate analysis, miRNAs were dichotomized as low or high using the ≤median vs. > median, 1st tercile vs. tercile 2-3, and 1st quartile vs. quartile 2-4 of its relative expression. For multivariate analysis, the miRNA dichotomization with the lowest P value from univariate analysis was used (see this table part [a] and [b]). The multivariate analysis used Breslow likelihood with a stepwise Backward removal of P values >0.15 (Statistica software v.14, Tibco).<br>CI stands for Confidential Interval.				

Supplementary Table 6a: Univariate Cox regression analyses of OS in validation (SWOG 0016) cohort (n=92).

Univariate Cox Proportional Hazard Regression:

**miR-29a**	**P value**	**Hazard Ratio (HR)**	**95% HR Lower CI**	**95% HR Upper CI**
miR-29a levels <median	0.094	0.5	0.22	1.13
miR-29a levels < 1st tercile	0.606	0.81	0.35	1.83
miR-29a levels < 1st quartile	0.795	0.89	0.37	2.14
**miR-29b**				
miR-29b levels <median	0.423	0.73	0.33	1.58
miR-29b levels < 1st tercile	0.387	0.68	0.28	1.63
miR-29b levels < 1st quartile	0.653	0.81	0.32	2.04
**miR-29c**				
miR-29c levels <median	0.371	1.43	0.65	3.14
miR-29c levels < 1st tercile	0.02	2.56	1.16	5.66
miR-29c levels < 1st quartile	0.018	2.76	1.19	6.38
FLIPI > 2	0.886	1.07	0.43	2.67
Age > 60 years	0.225	1.63	0.74	3.6
B symptoms present	0.139	0.48	0.18	1.27
LDH elevated	0.079	0.38	0.13	1.12
Hemoglobin <120 g/L	0.583	0.67	0.16	2.83

OS calculated from diagnosis as biopsy was performed at diagnosis. Ann Arbor Stage III-IV not used in the analysis due to only 3 patients having Stage 0-2.

For univariate analysis. miRNAs were dichotomized as low or high using the ≤median vs. > median. 1st tercile vs. tercile 2-3.

CI stands for Confidential Interval.

**Supplementary Table 6b:** Univariate Cox regression analyses of PFS for FL in validation (SWOG 0016) cohort (n=92).				
Univariate Cox Proportional Hazard Regression:				
**miR-29a**	**P value**	**Hazard Ratio (HR)**	**95% HR Lower CI**	**95% HR Upper CI**
miR-29a levels <median	0.19	1.45	0.83	2.53
miR-29a levels < 1st tercile	0.234	1.4	0.8	2.43
miR-29a levels < 1st quartile	0.25	1.41	0.79	2.51
**miR-29b**				
miR-29b levels <median	0.56	1.18	0.68	2.05
miR-29b levels < 1st tercile	0.269	1.37	0.78	2.41
miR-29b levels < 1st quartile	0.778	0.91	0.49	1.72
**miR-29c**				
miR-29c levels <median	0.289	1.35	0.78	2.35
miR-29c levels < 1st tercile	0.977	1.01	0.56	1.81
miR-29c levels < 1st quartile	0.381	0.73	0.37	1.47
FLIPI > 2	0.284	1.4	0.76	2.59
Age > 60 years	0.501	1.22	0.68	2.18
B symptoms present	0.216	0.68	0.37	1.26
LDH elevated	0.666	0.88	0.48	1.59
Hemoglobin <120 g/L	0.554	1.32	0.52	3.34
The PFS was calculated from the initiation of therapy. Ann Arbor Stage III-IV not used in the analysis due to only 3 patients having Stage 0-2.				
For univariate analysis. miRNAs were dichotomized as low or high using the ≤median vs. > median. 1st tercile vs. tercile 2-3.				
CI stands for Confidential Interval.				

**Supplementary Table 6c:** Multivariate Cox regression analyses of OS in validation (SWOG 0016) cohort (n=92). Multivariate analysis included FLIPI, Age, B symptoms, LDH. Hemoglobin and miRNA levels (dichotomisation selected from univariate analyses based on lowest P-value).				
Multivariate Cox Proportional Hazard Regression:				
**miR-29c**	**P value**	**Hazard Ratio (HR)**	**95% HR Lower CI**	**95% HR Upper CI**
LDH elevated	0.068	3.13	0.92	10.68
miR-29c levels	0.033	2.68	1.08	6.66
OS calculated from diagnosis as biopsy was performed at diagnosis. Ann Arbor Stage III-IV not used in the analysis due to only 3 patients having Stage 0-2.				
For univariate analysis, miRNAs were dichotomized as low or high using the ≤median vs. > median. 1st tercile vs. tercile 2-3. and 1st quartile vs. quartile 2-4 of its relative expression. For multivariate analysis. the miRNA dichotomization with the lowest P value from univariate analysis was used (see this table part [a] and [b]). The multivariate analysis used Breslow likelihood with a stepwise Backward removal of P values >0.15 (Statistica software v.14. Tibco).				
CI stands for Confidential Interval.				

References

1 Mraz M, Malinova K, Mayer J, Pospisilova S. MicroRNA isolation and stability in stored RNA samples. *Biochem Biophys Res Commun* 2009; **390**: 1–4.

2 Babraham Bioinformatics - FastQC A Quality Control tool for High Throughput Sequence Data. https://www.bioinformatics.babraham.ac.uk/projects/fastqc/ (accessed 9 June2023).

3 Martin M. Cutadapt removes adapter sequences from high-throughput sequencing reads. *EMBnet.journal* 2011; **17**: 10.

4 Pantano L, Estivill X, Martí E. SeqBuster, a bioinformatic tool for the processing and analysis of small RNAs datasets, reveals ubiquitous miRNA modifications in human embryonic cells. *Nucleic Acids Res* 2010; **38**: e34–e34.

5 Love MI, Huber W, Anders S. Moderated estimation of fold change and dispersion for RNA-seq data with DESeq2. *Genome Biol* 2014; **15**: 550.

6 Leggett RM, Ramirez-Gonzalez RH, Clavijo BJ, Waite D, Davey RP. Sequencing quality assessment tools to enable data-driven informatics for high throughput genomics. *Front Genet* 2013; **4**. doi:10.3389/fgene.2013.00288.

7 Bolger AM, Lohse M, Usadel B. Trimmomatic: a flexible trimmer for Illumina sequence data. *Bioinformatics* 2014; **30**: 2114–2120.

8 Smith T, Heger A, Sudbery I. UMI-tools: modeling sequencing errors in Unique Molecular Identifiers to improve quantification accuracy. *Genome Res* 2017; **27**: 491–499.

9 Dobin A, Davis CA, Schlesinger F, Drenkow J, Zaleski C, Jha S *et al.* STAR: ultrafast universal RNA-seq aligner. *Bioinformatics* 2013; **29**: 15–21.

10 Wang L, Wang S, Li W. RSeQC: quality control of RNA-seq experiments. *Bioinformatics* 2012; **28**: 2184–2185.

11 Okonechnikov K, Conesa A, García-Alcalde F. Qualimap 2: advanced multi-sample quality control for high-throughput sequencing data. *Bioinformatics* 2016; **32**: 292–294.

12 Liao Y, Smyth GK, Shi W. featureCounts: an efficient general purpose program for assigning sequence reads to genomic features. *Bioinformatics* 2014; **30**: 923–930.

13 Seda V, Vojackova E, Ondrisova L, Kostalova L, Sharma S, Loja T *et al.* FoxO1-GAB1 axis regulates homing capacity and tonic AKT activity in chronic lymphocytic leukemia. *Blood* 2021; **138**: 758–772.

14 Hoferkova E, Kadakova S, Mraz M. In Vitro and In Vivo Models of CLL-T Cell Interactions: Implications for Drug Testing. *Cancers* 2022; **14**: 3087.

15 Chang T-C, Yu D, Lee Y-S, Wentzel EA, Arking DE, West KM *et al.* Widespread microRNA repression by Myc contributes to tumorigenesis. *Nat Genet* 2008; **40**: 43–50.

16 Schindelin J, Arganda-Carreras I, Frise E, Kaynig V, Longair M, Pietzsch T *et al.* Fiji: an open-source platform for biological-image analysis. *Nat Methods* 2012; **9**: 676–682.

17 Mootha VK, Lindgren CM, Eriksson K-F, Subramanian A, Sihag S, Lehar J *et al.* PGC-1α-responsive genes involved in oxidative phosphorylation are coordinately downregulated in human diabetes. *Nat Genet* 2003; **34**: 267–273.

18 Subramanian A, Tamayo P, Mootha VK, Mukherjee S, Ebert BL, Gillette MA *et al.* Gene set enrichment analysis: A knowledge-based approach for interpreting genome-wide expression profiles. *Proc Natl Acad Sci* 2005; **102**: 15545–15550.

19 Parsa S, Ortega-Molina A, Ying H-Y, Jiang M, Teater M, Wang J *et al.* The serine hydroxymethyltransferase-2 (SHMT2) initiates lymphoma development through epigenetic tumor suppressor silencing. *Nat Cancer* 2020; **1**: 653–664.

20 Stuart T, Butler A, Hoffman P, Hafemeister C, Papalexi E, Mauck WM *et al.* Comprehensive Integration of Single-Cell Data. *Cell* 2019; **177**: 1888-1902.e21.

21 Roider T, Seufert J, Uvarovskii A, Frauhammer F, Bordas M, Abedpour N *et al.* Dissecting intratumor heterogeneity of nodal B cell lymphomas on the transcriptional, genetic, and drug response level. Cancer Biology, 2019 doi:10.1101/850438.

22 Alvarez MJ, Shen Y, Giorgi FM, Lachmann A, Ding BB, Ye BH *et al.* Functional characterization of somatic mutations in cancer using network-based inference of protein activity. *Nat Genet* 2016; **48**: 838–847.

23 Badia-i-Mompel P, Vélez Santiago J, Braunger J, Geiss C, Dimitrov D, Müller-Dott S *et al.* decoupleR: ensemble of computational methods to infer biological activities from omics data. *Bioinforma Adv* 2022; **2**: vbac016.

24 Garcia-Alonso L, Holland CH, Ibrahim MM, Turei D, Saez-Rodriguez J. Benchmark and integration of resources for the estimation of human transcription factor activities. *Genome Res* 2019; **29**: 1363–1375.

25 Schubert M, Klinger B, Klünemann M, Sieber A, Uhlitz F, Sauer S *et al.* Perturbation-response genes reveal signaling footprints in cancer gene expression. *Nat Commun* 2018; **9**: 20.

26 Browaeys R, Saelens W, Saeys Y. NicheNet: modeling intercellular communication by linking ligands to target genes. *Nat Methods* 2020; **17**: 159–162.

27 Liu A, Trairatphisan P, Gjerga E, Didangelos A, Barratt J, Saez-Rodriguez J. From expression footprints to causal pathways: contextualizing large signaling networks with CARNIVAL. *Npj Syst Biol Appl* 2019; **5**: 40.

28 Türei D, Korcsmáros T, Saez-Rodriguez J. OmniPath: guidelines and gateway for literature-curated signaling pathway resources. *Nat Methods* 2016; **13**: 966–967.

29 Steen CB, Liu CL, Alizadeh AA, Newman AM. Profiling Cell Type Abundance and Expression in Bulk Tissues with CIBERSORTx. In: Kidder BL (ed). *Stem Cell Transcriptional Networks*. Springer US: New York, NY, 2020, pp 135–157.

30 Newman AM, Steen CB, Liu CL, Gentles AJ, Chaudhuri AA, Scherer F *et al.* Determining cell type abundance and expression from bulk tissues with digital cytometry. *Nat Biotechnol* 2019; **37**: 773–782.

31 Cari, De Rosa, Petrillo, Migliorati, Nocentini, Riccardi. Identification of 15 T Cell Restricted Genes Evaluates T Cell Infiltration of Human Healthy Tissues and Cancers and Shows Prognostic and Predictive Potential. *Int J Mol Sci* 2019; **20**: 5242.

32 Szabo PA, Levitin HM, Miron M, Snyder ME, Senda T, Yuan J *et al.* Single-cell transcriptomics of human T cells reveals tissue and activation signatures in health and disease. *Nat Commun* 2019; **10**: 4706.

33 Babicki S, Arndt D, Marcu A, Liang Y, Grant JR, Maciejewski A *et al.* Heatmapper: web-enabled heat mapping for all. *Nucleic Acids Res* 2016; **44**: W147–W153.

34 Huet S, Tesson B, Jais J-P, Feldman AL, Magnano L, Thomas E *et al.* A gene-expression profiling score for prediction of outcome in patients with follicular lymphoma: a retrospective training and validation analysis in three international cohorts. *Lancet Oncol* 2018; **19**: 549–561.

35 Sharma S, Pavlasova GM, Seda V, Cerna KA, Vojackova E, Filip D *et al.* *miR-29* modulates CD40 signaling in chronic lymphocytic leukemia by targeting TRAF4: an axis affected by BCR inhibitors. *Blood* 2021; **137**: 2481–2494.

36 Liang M, Zhang C, Yang Y, Cui Q, Zhang J, Cui C. TransmiR v3.0: an updated transcription factor-microRNA regulation database. *Nucleic Acids Res* 2025; **53**: D318–D323.

37 Press OW, Unger JM, Rimsza LM, Friedberg JW, LeBlanc M, Czuczman MS *et al.* Phase III Randomized Intergroup Trial of CHOP Plus Rituximab Compared With CHOP Chemotherapy Plus ^{131} Iodine-Tositumomab for Previously Untreated Follicular Non-Hodgkin Lymphoma: SWOG S0016. *J Clin Oncol* 2013; **31**: 314–320.
